# Aberrant stromal tissue factor localisation and mycolactone-driven vascular dysfunction, exacerbated by IL-1β, are linked to fibrin formation in Buruli ulcer lesions

**DOI:** 10.1371/journal.ppat.1010280

**Published:** 2022-01-31

**Authors:** Louise Tzung-Harn Hsieh, Scott J. Dos Santos, Belinda S. Hall, Joy Ogbechi, Aloysius D. Loglo, Francisco Javier Salguero, Marie-Thérèse Ruf, Gerd Pluschke, Rachel E. Simmonds

**Affiliations:** 1 Department of Microbial Sciences, School of Bioscience and Medicine, University of Surrey, Guildford, United Kingdom; 2 UK Health Security Agency, Manor Farm Road, Porton Down, Salisbury, United Kingdom; 3 Swiss Tropical and Public Health Institute, Basel, Switzerland; 4 University of Basel, Basel, Switzerland; University of Washington, UNITED STATES

## Abstract

Buruli ulcer (BU) is a neglected tropical disease caused by subcutaneous infection with *Mycobacterium ulcerans* and its exotoxin mycolactone. BU displays coagulative necrosis and widespread fibrin deposition in affected skin tissues. Despite this, the role of the vasculature in BU pathogenesis remains almost completely unexplored. We hypothesise that fibrin-driven ischemia can be an ‘indirect’ route to mycolactone-dependent tissue necrosis by a mechanism involving vascular dysfunction. Here, we tracked >900 vessels within contiguous tissue sections from eight BU patient biopsies. Our aim was to evaluate their vascular and coagulation biomarker phenotype and explore potential links to fibrin deposition. We also integrated this with our understanding of mycolactone’s mechanism of action at Sec61 and its impact on proteins involved in maintaining normal vascular function. Our findings showed that endothelial cell dysfunction is common in skin tissue adjacent to necrotic regions. There was little evidence of primary haemostasis, perhaps due to mycolactone-dependent depletion of endothelial von Willebrand factor. Instead, fibrin staining appeared to be linked to the extrinsic pathway activator, tissue factor (TF). There was significantly greater than expected fibrin staining around vessels that had TF staining within the stroma, and this correlated with the distance it extended from the vessel basement membrane. TF-induced fibrin deposition in these locations would require plasma proteins outside of vessels, therefore we investigated whether mycolactone could increase vascular permeability *in vitro*. This was indeed the case, and leakage was further exacerbated by IL-1β. Mycolactone caused the loss of endothelial adherens and tight junctions by the depletion of VE-cadherin, TIE-1, TIE-2 and JAM-C; all Sec61-dependent proteins. Taken together, our findings suggest that both vascular and lymphatic vessels in BU lesions become “leaky” during infection, due to the unique action of mycolactone, allowing TF-containing structures and plasma proteins into skin tissue, ultimately leading to local coagulopathy and tissue ischemia.

## Author summary

To date, the debilitating skin disease Buruli ulcer remains a public health concern and financial burden in low or middle-income countries, especially in tropical regions. Late diagnosis is frequent in remote areas, perhaps due to the painlessness of the disease. Hence patients often present with large, destructive opened ulcers leading to delayed wound closure or even lifelong disability. The infectious agent produces a toxin called mycolactone that drives the disease. We previously found evidence that the vascular system is disrupted by mycolactone in these lesions, and now we have further explored potential explanations for these findings by looking at the expression of vascular markers in BU. In a detailed analysis of patient skin punch biopsies, we identified distinct expression patterns of certain proteins and found that tissue factor, which initiates the so-called extrinsic pathway of blood clotting, is particularly important. Mycolactone is able to disrupt the barrier function of the endothelium, further aggravating the diseased phenotype, which may explain how clotting factors access the tissue. Altogether, such localised hypercoagulation in Buruli ulcer skin lesions may contribute to the development of the lesion.

## Introduction

Buruli ulcer (BU) is a neglected tropical disease found in over 30 countries world-wide resulting from a subcutaneous infection with *Mycobacterium ulcerans*, an opportunistic environmental pathogen. According to the World Health Organization (WHO), approximately 2700 new cases were reported globally in 2018 [1], which suggests case numbers are falling from a peak of 5,000–6,000 per year in 2004–2010. However, the actual number of cases is likely far higher, since many BU endemic countries do not report data to the WHO [2]. Approximately 13% of the 2018 cases were in Australia, where the state of Victoria [3] is seeing increasing and sustained case numbers. Hence Buruli ulcer (known as Bairnsdale ulcer in Australia) is a public health problem of world-wide concern.

Despite its relative rarity globally, BU is a devastating disease in communities with high endemicity, where the prevalence can be up to 77 cases per 10,000 population [2–4]. BU often presents late, due to the characteristic painlessness of the lesions and lack of other overt signs of infection such as fever and malaise. In these patients, necrotic skin ulcers and soft tissue destruction can extend up to 15% of body surface area. Even smaller lesions may cause lifelong disability [5]. While the infection can be effectively sterilised with dual anti-mycobacterial antibiotics [6], in some settings surgery is still performed, involving debridement of infected tissue with or without skin grafts and, in extreme cases, amputation [7]. The disease imposes a large socio-economic burden on endemic communities, as treatment can require long hospital stays for patients, many of whom are young teenagers. Indeed, wound-healing remains a critical issue in BU as it can take more than 12 months, even in high resource settings [6]. Therefore, to achieve the long-term goal of reducing the disease burden and serious sequelae, there is a real need for a better understanding of BU pathogenesis.

Clinically, BU presents in various forms: nodules, plaques, ulcers, and oedema [4]. Importantly, these are not inevitably progressive “stages” of disease. For example, nodules (or papules) are palpable lumps under the skin that already have a core of necrotic tissue containing clusters of acid-fast bacilli (AFB) surrounded by healthy tissue [8,9]. On the other hand, plaques consist of wide areas of necrotic skin tissue under an intact dermis [10] giving rise to a characteristic indurated presentation. Oedematous forms present with substantial diffuse swelling, which can be mistaken for cellulitis clinically, although oedema due to BU is discriminated by a lack of pain at the affected site and absence of fever. Nodules, plaques and oedematous forms all have the potential to ulcerate, and BU ulcers are usually surrounded by an area of plaque.

Histopathological analysis of BU patient biopsies reveals the unique features of *M*. *ulcerans* infections [11], even when compared to other mycobacterial skin infections such as “fishtank granuloma” (*M*. *marinum* infection), cutaneous tuberculosis or leprosy. The infected macrophages within a granuloma structure typical of these infections are rarely seen in BU except during antibiotic treatment or in late-presenting chronic disease [12]. Instead, the bacteria are predominantly found in clusters of extracellular bacilli, frequently at the base of the subcutis. Importantly, these clusters are not necessarily seen in every section of a biopsy sample of a BU patient [9,12,13]. The characteristic features of BU lesions are coagulative necrosis (where the cellular architecture remains intact but anucleated cells are prevalent) along with “fat cell ghosts” and epidermal hyperplasia. Necrosis may be seen extending away from the AFB [11,14], a feature that presented the very earliest clue that BU pathogenesis is largely driven by a secreted diffusible toxin [15–17], later identified as mycolactone [14]. Mycolactone is also immunosuppressive, explaining the relative paucity of leukocytic infiltration close to site of infection [11,18]. Instead, a belt of infiltrating cells including neutrophils, macrophages and lymphocytes, surrounds the necrotic core, some distance from the bacteria [9,19]. However, it is important to note that a technique to determine mycolactone’s spatial distribution within tissue currently eludes the field.

Much is now known about how mycolactone mediates its pathogenic effects. Mycolactone is a lipid-like compound [14], produced by polyketide synthases encoded on the *M*. *ulcerans* megaplasmid, pMUM [20]. Mycolactone targets a vital cellular process found in all nucleated host cells: the transport of proteins into the endoplasmic reticulum (ER) by a the heterotrimeric Sec61 translocon [21]. Mycolactone inhibits the co-translational translocation of many secretory and membrane proteins via the Sec61 complex [22–25]. This discovery has been transformational in our understanding of BU pathogenesis, since it explains both the direct cytotoxicity of mycolactone (due to the cellular stress invoked) and the immunosuppression (since many immune mediators, including inflammatory cytokines, are Sec61-dependent secretory or membrane proteins).

We still know relatively little about the effect mycolactone has on other local physiological pathways within infected skin. Due to the painlessness of the lesions, some research has focussed on its effects on neurons and neuronal cells [26–28]. Another key candidate, and the topic of the current work, is the vascular system, because coagulative necrosis is commonly associated with ischemia due to blood vessel dysfunction. Moreover, fibrin deposition is a common feature of BU lesions, reported as long ago as the mid-20^th^ Century, and this phenotype is strongly correlated with tissue necrosis and delayed wound healing when sustained [29]. Finally, the chronic unhealing ulcers seen in BU, which may even persist after sterilisation with antibiotics, are reminiscent of ulcers seen in other diseases with vascular complications such as in patients with diabetes mellitus [30]. However, despite this, vessel function in BU remains almost completely unexplored.

A healthy vasculature cannot be separated from the function of endothelial cells, a heterogeneous yet continuous monolayer that lines both arterial and venous blood vessels, as well as the microvasculature and lymphatics [31,32]. Once considered to be a merely a ‘lining’ for blood vessels, it is now clear that the endothelium is a dynamic regulatory organ, forming a semi-selective barrier that controls the passage of small molecules and white blood cells into and out of the bloodstream, and can respond to wide range of stimuli that alter tissue homeostasis [33,34].

A central function of endothelial cells is to maintain blood fluidity under normal conditions, and to particate in the response to vessel damage. This blood clotting process involves a variety of blood-borne proteins (eg coagulation factors) and cell-derived components (such as platelets and cell-derived microparticles). Endothelial cells express von Willebrand factor (vWF), stored within organelles called Weibel-Palade bodies (WBPs) [35]. Upon exposure to so-called secretagogues, such as the coagulation factor thrombin, these are exocytosed, leading to the formation of multimeric strings of vWF molecules whose function is to facilitate platelet binding to collagen exposed at the injured vessel wall [36]. Formation of platelet plugs is known as ‘primary haemostasis’ [32,34].

Secondarily to platelet plug formation, coagulation reinforces the plug by producing a fibrin mesh to hold it in place. Two coagulation pathways are involved in fibrin generation, known as the intrinsic and extrinsic pathways. The intrinsic pathway, or contact activation, involves autoactivation of factor XII on a negatively-charged surface [37]. The extrinsic pathway is initiated when tissue factor (TF) binds to factor VII/VIIa forming a complex that activates factor Xa. Both pathways ultimately lead to rapid amplification of thrombin formation and processing of fibrinogen to fibrin [38]. In disease states, fibrin accumulation within vessels can lead to thrombosis, where the circulation of blood is impeded by physical obstruction due to the fibrin clot. As a result of the serious consequences of uncontrolled coagulation, the endothelium expresses a variety of anticoagulant proteins that regulate thrombin generation [34]. These include thrombomodulin (sometimes abbreviated to TM) and the endothelial cell protein C receptor (EPCR) that activate the protein C anticoagulant pathway [34,39], tissue factor pathway inhibitor (TFPI) that supresses the TF-factor VIIa complex and factor Xa activity [40], and heparan sulphate proteoglycans within the glycocalyx that bind antithrombin to directly inhibit thrombin activity [41].

In the only study to date on the effect of mycolactone on endothelial cells and the vasculature in BU, we showed that mycolactone depletes the Sec61-dependent proteins vascular endothelial cadherin (VE-cadherin), TM and EPCR in primary human dermal microvascular endothelial cells (HDMECs) [42]. This profoundly reduces the ability of mycolactone-exposed endothelial cells to activate protein C, implying that endothelial cells may display a pro-coagulant phenotype *in vivo*. We also showed reduced TM expression in BU patient skin biopsies, suggesting endothelial cell dysfunction in the diseased tissue [42]. This study described a correlation between thrombomodulin depletion and fibrin abundance, but had the limitation that we were unable to track individual vessels. Furthermore, we found that mycolactone had no effect on platelet function *in vitro*, leaving open the question of whether primary haemostasis occurs in BU skin lesions. Nevertheless, our findings led us to hypothesise that mycolactone may provide both ‘direct’ and ‘indirect’ routes to necrosis *in vivo*. In our model, the direct effect is slow, driven by the cytotoxicity that follows Sec61 inhibition, which takes several days [22], The indirect effect results from fibrin deposition after rapid loss of Sec61-dependent coagulation modulators, causing mycolactone-induced ischaemia.

The overall aim of the current work was to investigate the hypothesis that the ‘indirect’ route to tissue necrosis involving vascular dysfunction is involved in BU pathogenesis. This was achieved by carrying out detailed analysis of BU patient punch biopsy samples through a series of contiguous sections. The primary aim was to identify and track a large number of vessels and evaluate their phenotype with regard to vascular and coagulation biomarkers to understand which may be linked to fibrin deposition. The secondary aim was to integrate this with our understanding of mycolactone’s mechanism of action at Sec61 and its impact on proteins involved in maintaining normal vascular function, using *in vitro* studies on mycolactone-exposed primary endothelial cells to seek supporting evidence for processes that may drive the clinical observations.

The clinical samples generated a complex dataset, in which 908 individual vessels were tracked and analysed relative to staining for fibrin using an antibody that discriminates from its precursor, fibrinogen. The data will be presented in the following order. First we provide a more in-depth analysis of CD31 and thrombomodulin loss from endothelial cells. We then consider whether there is any evidence primary haemostasis in the lesions, on the basis of staining for endothelial vWF and CD61 (a marker for Integrin β3 expressed on platelets). Finally, we consider the staining pattern for TF, the extrinsic factor activator, which was the only marker analysed that correlated with fibrin deposition. Supporting *in vitro* data from primary endothelial cells is presented at relevant points throughout, and culminates in vascular permeability assays. Hence, mycolactone-dependent changes in endothelial cell morphology leading to increased vascular permeability may underpin the vasculopathy and fibrin deposition seen in BU.

## Results

In order to better understand how the vascular and coagulation systems are involved in BU pathogenesis, particularly in the fibrin deposition that we hypothesise may be linked to tissue necrosis, we analysed nine contiguous sections from each of eight skin punch biopsies taken from different, untreated, laboratory confirmed BU patients (6 with ulcerative lesions and 2 with plaques, covering 3 WHO categories; Table 1 and S1 Fig). In BU patients displaying ulcerated lesions, punch biopsies were taken 1 cm inside the outer margin of the induration surrounding the ulcer. Otherwise, punch biopsies were collected from the non-ulcerated centre of the lesion. The sections were stained by haematoxylin-eosin (H&E) and Ziehl-Neelsen, and immunohistochemistry for fibrin, thrombomodulin, CD31, smooth muscle actin (SMA), vWF, CD61 and TF was performed (S1 Fig). We chose to use the same biopsy samples as in our previous study [42] in order to facilitate a direct comparison between the same markers across these 4mm biopsy samples. This revealed that the pathology of Buruli ulcer is highly focal (S2A Fig), justifying the need to utilise contiguous sections within the same biopsy, in order to minimise drift and allow the tracking of individual vessels between the sections (S2B Fig). Importantly, while all the biopsies are positive for AFB in at least one section, in our contiguous section that was used for Ziehl-Neelsen staining, AFB were only detected in the sections from two of the patients (S2C Fig). Therefore, we do not report analysis of the coincidence of Ziehl-Neelsen staining further in the current work.

**Table 1:** Clinical features of analysed Buruli ulcer patients. All 8 patients had previously been confirmed to have Buruli ulcer [42]. Category 1: a single lesion < 5 cm in diameter; Category 2: a single lesion 5–15 cm in diameter; Category 3: a single lesion > 15 cm in diameter, multiple small lesions or facial lesions. Age is presented in median (with range) and the rest of data are n (%).

Age (years)		12 (7–70)
Type of lesion	Ulcerative	6 (75%)
	Plaque	2 (25%)
WHO category	1	2 (25%)
	2	4 (50%)
	3	2 (25%)
Location	Upper extremities	4 (50%)
	Lower extremities	4 (50%)

As expected, all the punch biopsies displayed widespread necrosis, which is a defining feature of BU thought be due to the diffusibility of mycolactone. Highly necrotic areas are not well-suited to immunohistochemical analysis, since the pathological process has passed the endpoint, and such regions can be associated with higher background staining. However, all biopsies also contained at least one area adjacent to the necrotic regions where a pathologist could identify few sub-macroscopic signs of coagulative necrosis, including blood vessels where nuclei were still evident under H&E staining, and which had reasonable staining for the well-characterised vessel markers SMA and CD31 as the submacroscopic level. We therefore focussed our analysis only on vessels identified in these pathologist-defined “least necrotic” regions (outlined in red in S1 Fig, nine in the dermis, one in the subcutis; two patients contained two such areas), which represented 17.6–48.2% (median 24.3%) of the total biopsy area. In order to facilitate an unbiased analysis, we performed quantitative analysis on all identifiable vessels within these regions, regardless of whether they were later revealed to contain localised microscopic signs of necrosis, such as anucleated cells.

### Vasculopathy is common in Buruli ulcer lesions

Our first step when analysing this complex dataset was to revisit the analysis of the well-known perivascular and/or endothelial cell markers CD31, SMA and thrombomodulin, previously performed in non-contiguous sections without vessel tracking [42]. Since we showed both thrombomodulin and, to a lesser extent, CD31 are depleted by mycolactone exposure we took a conservative approach and identified vessels which stained positively for SMA, or at least one of thrombomodulin and/or CD31. Using this approach, a total of 908 vessels were tracked in the eight patient skin biopsies (median of 92.5 vessels per patient sample, range 18–253).

**Fig 1:**
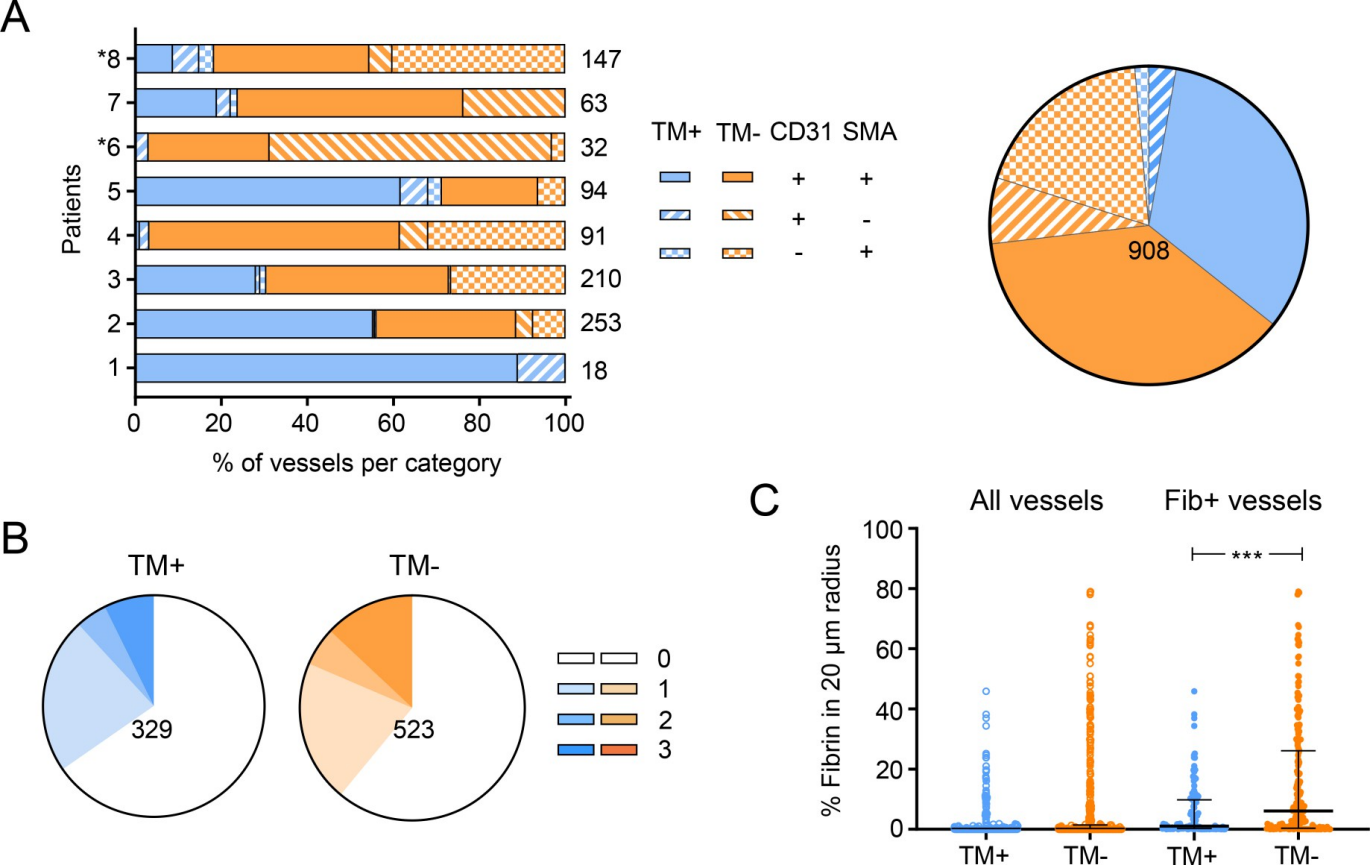
Thrombomodulin expression and fibrin deposition in and around blood vessels. **A.** Vessels were tracked by the endothelial cell marker CD31 and perivascular cell marker SMA, analysed and categorised according to the expression of TM, CD31 and SMA. The number of vessels in each category per patient was expressed as a percentage of the total number of the vessels counted for that representative area (listed right). Patients displaying plaque lesions are indicated with asterisks. The pie chart to the right shows the overall distribution of each category. **B and C.** The degree of fibrin deposition surrounding tracked vessels in A. **B** represents the distribution of fibrin scores of thrombomodulin-positive (TM^+^) and thrombomodulin-negative (TM^-^) vessels. Increasing fibrin scores corresponding to increasing extension of fibrin staining and are represented by stronger colour (0; no fibrin staining with 20 μm, 1–3 are fibrin staining in a <20, 20–30 and >30 μm radius, respectively). The total number of the vessels analysed is shown. **C.** The percentage of the area within 20 μm of each tracked vessel that stained positively for fibrin (TM^-^ or TM^-^) was determined using Nikon Elements software, and is presented for all and fibrin-positive (Fib^+^) vessels. Data for individual vessels as well as median and interquartile range is shown. ***; *P* < 0.001.

When vessels were categorised according to the expression of SMA, CD31 and thrombomodulin at any level of positive staining, thrombomodulin presence was found to vary widely, regardless of the lesion type or WHO Category, closely matching our previous analysis. This gave us sufficient confidence to continue, despite an overall lower intensity of staining for thrombomodulin in these sections [42], which made identification of TM^-^ vessels more difficult. All the vessels of one patient were TM^+^ (Fig 1A), although for this patient the least-necrotic region was very small and contained only 18 vessels (S1 Fig). In contrast, two patients had few TM^+^ vessels (~3%), while other patients’ samples showed intermediate degrees of positive staining (Fig 1A). Overall, only 299 out of 908 (32.9%) vessels showed a normal staining pattern (TM^+^ CD31^+^ SMA^+^). The remaining vessels lacked one or more of the expected markers, with the most common phenotypes being TM^-^ CD31^+^ SMA^+^ (37.4%) and TM^-^ CD31^-^ SMA^+^ (18.7%). Some of the singly-positive SMA structures could arguably be sweat glands with vessel-like morphology, but since many of these proved positive for the endothelial cell-specific vWF (S1 Table, and see below) only 60 vessels (6.6% of the 908 analysed) remained questionable. Hence, even if a proportion of singly-positive SMA structures are glands, the data still suggest a general vasculopathy in BU lesions that may conceivably precede the emergence of coagulative necrosis.

In order to understand whether the previously identified association between thrombomodulin depletion and fibrin deposition [42] is functionally linked, fibrin deposition was scored for each vessel according to distance of fibrin staining from the vessel, where a score of 0 indicated no fibrin staining, and scores of 1–3 indicated fibrin staining in a <20, 20–30 and >30 μm radius of the vessel, respectively. Here, 852 vessels were trackable, since not all vessels identified above could be found in the fibrin-stained section. As reported previously [42], fibrin staining was seen across the biopsies, varying in intensity and coverage for each patient (S1 Fig). In the current analysis, fibrin deposition was found in tissue surrounding 298/852 (35.0%) of the tracked vessels. On the other hand, fibrin staining within blood vessels was rarely seen (in a maximum 20 vessels).

Analysis of the fibrin scores for 329 TM^+^ and 523 TM^-^ vessels showed thrombomodulin expression had no overall impact on fibrin deposition within tissue (chi^2^ vs normal staining pattern *P* = 0.2007, Fig 1B). Hence, thrombomodulin depletion itself does not seem to be a good predictor of local fibrin deposition. However, while most vessels were fibrin-negative, in vessels lacking thrombomodulin expression the proportion with the highest fibrin score was increased compared to TM^+^ vessels (13.0 vs. 7.3%, Fig 1B). Moreover, the maximum percentage of the area positively staining for fibrin within 20 μm of vessels was higher for TM^-^ vs TM^+^ vessels (79.0 vs. 45.8%, Fig 1C), suggesting the loss may aggravate fibrin formation in certain subgroup(s) of vessels. This is borne out when the analysis was repeated for vessels that had a fibrin score of ≥1, where the percentage of the area positively staining for fibrin within 20 μm reached statistical significance (Fig 1C).

In conclusion, the vast majority of endothelial cells in the majority of BU patients have evidence of vasculopathy, as they no longer express at least one of the constitutive endothelial cell markers. In addition, while fibrin deposition surrounding vessels is quite common, the loss of thrombomodulin (leading to a local procoagulant phenotype within affected vessels due to an inability to activate the protein C anticoagulant pathway) cannot not explain all the fibrin disposition seen in BU patient lesions.

### Poor evidence for primary haemostasis in BU, perhaps due to mycolactone-dependent depletion of von Willebrand factor from endothelial cells

The next analysis we performed was to investigate whether fibrin deposition was linked to primary haemostasis, by examining the expression and location of markers involved in this process [34]. Activated platelets express platelet glycoprotein IIb/IIIa (CD41/CD61), as their main receptor for vWF and fibrinogen, and here we examined staining for CD61, also known as integrin β3 [43]. Since we have previously shown that platelet activation is not directly affected by mycolactone [42], abundant CD61 staining in vessels would indicate activated platelets had formed a platelet plug by binding to collagen and vWF, suggesting primary haemostasis in BU lesions. Note that it is widely accepted that CD61 does not stain healthy tissue, being a marker specific for activated platelets that is not constitutively expressed by any cells in healthy skin.

**Fig 2:**
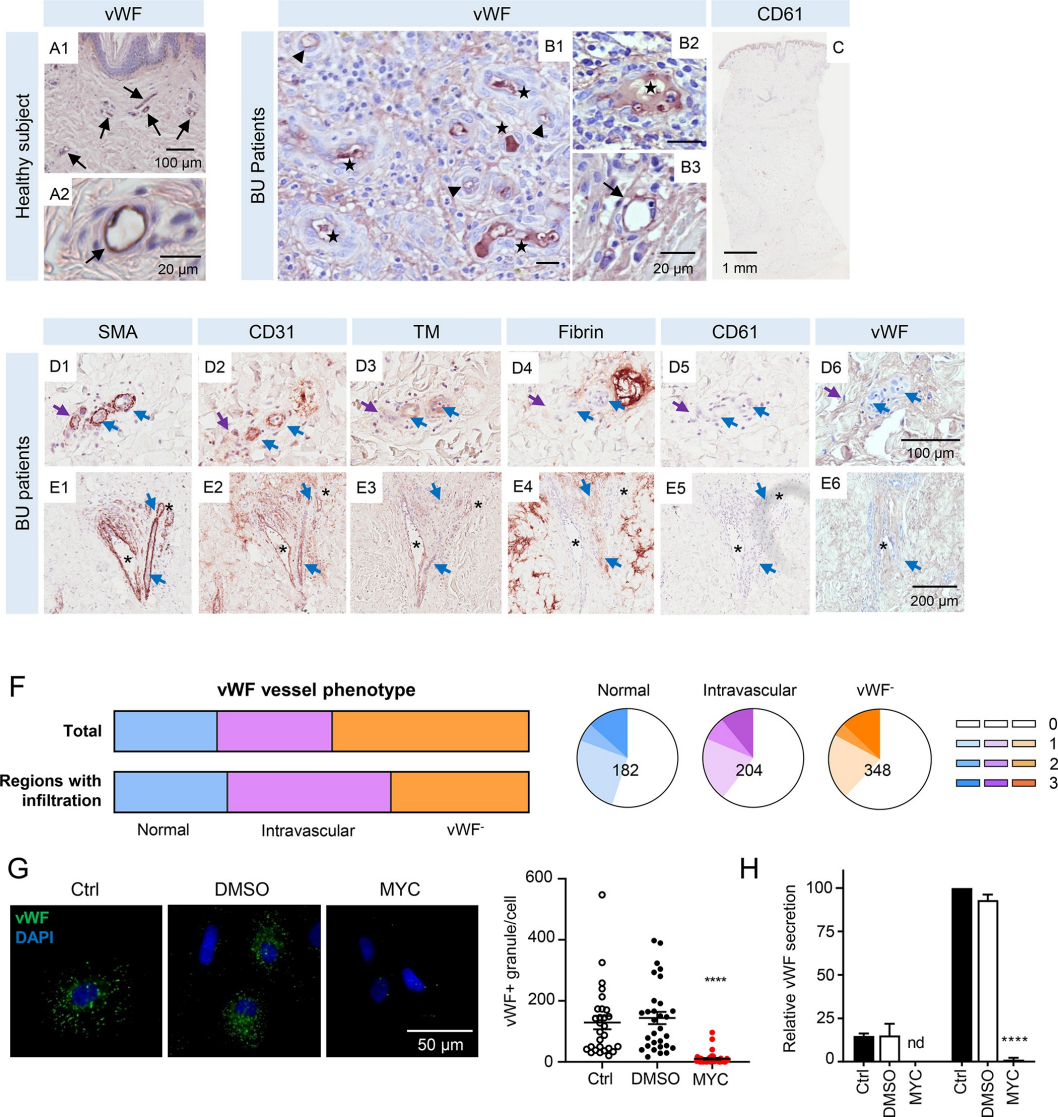
Endothelial von Willebrand factor is downregulated in response to mycolactone and redistributes in BU lesions. **A-E.** Histological sections stained with antibodies against vWF (A1-2, B1-3, D6 and E6), platelet glycoprotein CD61 (C, D5 and E5), SMA (D1 and E1), CD31 (D2 and E2), TM (D3 and E3) and fibrin (D4 and E4) (positive staining in brown colour) and counterstained with haematoxylin in a healthy subject (A1-2) and BU patients (B, C, D and E). Scale bars as indicated. **A.** Examples of normal vessel staining for vWF (black arrows). **B.** Examples of different phenotypes of abnormal vWF staining that were observed; black arrowheads (reduced expression), black stars (vessels displaying intravascular vWF staining). **C.** Overview of CD61 staining in BU patient 5. **D and E.** Examples of vessels with different phenotypes in BU punch biopsy samples are indicated as follows; purple arrows are SMA^+^CD31^-^TM^-^, blue arrows are SMA^+^CD31^+^TM^-^, and asterisks are SMA^+^CD31^+^TM^+^ vessels. **F.** The overall distribution of vWF staining patterns in all patients (top panel) and vessels in immune cell-infiltrated regions (lower panel). The distribution of fibrin scores per vWF staining pattern (i.e. normal, intravascular and vWF^-^) is represented by pie charts with the total vessel number shown. Increasing fibrin scores corresponding to increasing extension of fibrin staining are represented by darker colour (0; no fibrin staining with 20 μm, 1–3 are fibrin staining in a <20, 20–30 and >30 μm radius, respectively). **G and H.** Primary endothelial cells were treated with 10 ng/mL of mycolactone (MYC), 0.02% DMSO or untreated (Ctrl) for 24 hours. **G.** HUVECs were fixed, permeabilised and immunostained with anti-vWF antibody. vWF-containing granules are shown in green and nuclei stained with DAPI (blue). Scale bar = 50 μm. Scatter plot showing vWF-positive granules per cell in each condition of three independent experiments. ****; *P* < 0.0001. **H.** HDMECs were treated with 2 U/mL thrombin to induce exocytosis of Weibel-Palade bodies. The concentration of vWF in supernatants was quantified by ELISA. Values represent the mean of three independent experiments ± SEM. nd, not detected. ****; *P* < 0.0001.

Surprisingly, considering the wide abundance of fibrin in the lesions, very little CD61 staining was seen in any of the eight BU samples (Figs 2C and S1). In vessels that were positive for of least one of SMA, CD31 or thrombomodulin, none stained positively for CD61 (Figs 2D1-6 and 2E1-6), despite strong fibrin staining in the vicinity (Fig 2D4 and 2E4, blue and purple arrows).

To find an explanation for this surprising lack of platelet activation, we incorporated our understanding of mycolactone’s blockade of Sec61-dependent protein production. As mentioned in the introduction, vWF is the primary component of WBPs, secretory granules released from endothelial cells in response to activation or injury [44] in order to capture activated platelets. Since vWF is a Sec61-dependent secretory protein, we postulated its mycolactone-dependent depletion from endothelial cells might explain the lack of CD61/platelet staining.

In healthy skin, vWF was present in endothelial cells lining blood vessels as expected (Figs 2A1-2 and S3A) [45]. As others have observed [46,47], there is a relatively high stromal background for vWF staining due to its presence in serum. By contrast, in the BU biopsies, detection of endothelial vWF was frequently reduced (Fig 2B1, black arrowheads) or staining was seen in the intravascular space instead (Fig 2B1-2, black stars); rarely, vWF remained detectable in the endothelium (Fig 2B3, black arrow). Indeed, of 734 trackable vessels, only 24.8% retained a normal expression pattern, with the endothelial monolayer staining positive for vWF (Fig 2B3 and 2F upper panel). Instead, nearly half (47.4%) had completely lost vWF staining (vWF^-^) (Fig 2B1, 2D6, 2E6, and 2F upper panel). Intriguingly, 204 vessels (27.8%) displayed vWF staining within the lumen of microvessels, in the intravascular space (Fig 2B1-2 and 2F upper panel). This pattern of staining was particularly evident in areas that were heavily infiltrated with immune cells (S1 Fig, outlined in blue), representing 52.5% of such vessels (Fig 2F, lower panel). However, fibrin scores were similar across all three phenotypes of vWF expression in vessels (chi^2^ vs normal staining pattern *P* = 0.2882 and 0.1280 for intravascular and vWF^-^, respectively, Fig 2F), suggesting that none of these phenotypes are directly related to fibrin formation.

Supporting evidence for mycolactone-dependent vWF depletion was derived from *in vitro* studies examining its expression in cultured primary HDMECs and human umbilical vein endothelial cells (HUVECs) by immunofluorescence. First, we established that HUVECs and HDMECs have a similar IC_50_ for mycolactone after 5 days exposure (S3B Fig), and that, like HDMECs, HUVECs have minimally reduced viability at 24 hrs (S3C Fig). Although the number of vWF-positive granules (presumptive WBP) varied from cell to cell, these were greatly reduced after 24 hours exposure to mycolactone (Figs 2G and S3D). Furthermore, when we quantified vWF in conditioned medium from HDMECs by enzyme-linked immunosorbent assay (ELISA), both basal and thrombin-induced vWF secretion were almost completely abolished following mycolactone exposure (Fig 2H).

Taken together, our analysis of CD61 and vWF staining in BU patient samples and our *in vitro* studies on platelets [42] and vWF expression by endothelial cells suggest a minor role, if any, for primary haemostasis in BU lesion formation. Mycolactone’s inhibition of formation of vWF-containing WBPs may play a role here, since the endothelial cells lining vessels exposed to mycolactone are less likely to be able to produce vWF to capture activated platelets.

### Mycolactone reduces the expression of tissue factor pathway inhibitor in endothelial cells

Having ruled out a role for primary haemostasis, we considered the extrinsic pathway of coagulation activation. As we had found that the Sec61-dependent protein vWF was depleted from endothelial cells by mycolactone, we asked whether these cells might lack the critical regulator of this pathway, TFPI [34,40]. Our rationale was that TFPI is a signal peptide-containing protein that relies upon Sec61 for the production of both its isoforms. Full-length TFPI (isoform α) is a GPI-anchored protein, whereas isoform β lacks both the C-terminus and the third Kunitz-type inhibitory domain. TFPIβ is secreted but associates with the cell surface via glycosaminoglycan interactions. We therefore predicted that TFPI expression by endothelial cells would be sensitive to mycolactone.

**Fig 3:**
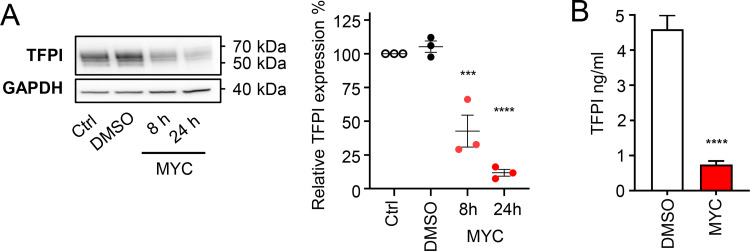
Mycolactone reduces the expression of tissue factor pathway inhibitor in endothelial cells. **A.** HDMECs exposed to 10 ng/mL of mycolactone (MYC) for the indicated times, 0.02% DMSO or untreated (Ctrl) were lysed and subjected to immunoblotting. TFPI immunoblot intensity was normalised according to GAPDH and untreated controls. Data from 3 independent experiments are presented (mean ± SEM). **B.** Supernatant was collected from cells treated as above for 24 hours, cell debris removed and TFPI was quantified by ELISA. Values represent the mean of three independent experiments ± SEM. ***, *P* < 0.001; ****, *P* < 0.0001.

Since TFPIα and β migrate with similar molecular mass by SDS-PAGE [48], we couldn’t distinguish between the isoforms expressed by HDMEC via immunoblotting (Fig 3A). However, in line with our hypothesis, we found that TFPI was rapidly depleted following mycolactone exposure with a decrease of 57.4% after 8 hours of mycolactone exposure and 88.3% after 24hours (Fig 3A). A similar decline was seen for non-cell-associated TFPI by ELISA of HDMEC conditioned medium, where TFPI levels were reduced from 4.59±0.18 to 0.74±0.13 ng/mL (Fig 3B). The loss of THPI suggests negative regulation of the extrinsic coagulation cascade may be compromised in BU.

### Extravascular tissue factor within BU lesions correlates to pathogenic fibrin deposition

**Fig 4:**
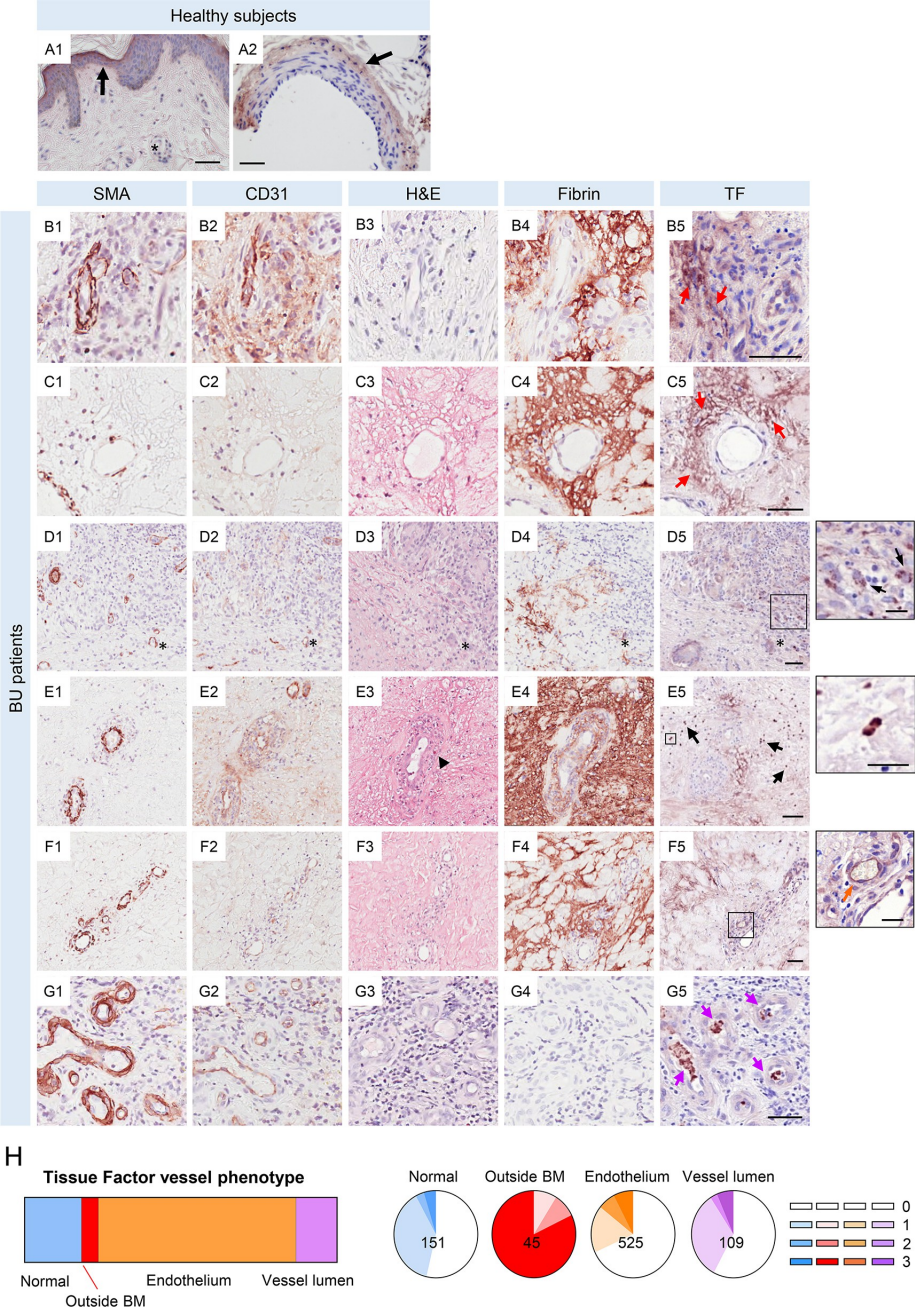
Tissue factor expression is altered in BU patient skin lesion. **A-G.** Histological sections were stained with anti-tissue factor (TF) antibody (A1-2, B5, C5, D5, E5, F5 and G5), anti-SMA antibody (B1, C1, D1, E1, F1 and G1), anti-CD31 antibody (B2, C2, D2, E2, F2 and G2), eosin (B3, C3, D3, E3, F3 and G3) or anti-fibrin antibody (B4, C4, D4, E4, F4 and G4) (positive staining in a brown colour) and counterstained with haematoxylin in healthy subjects (A) or in BU punch biopsy samples (B-G). Scale bar = 50 μm (20 μm in the crop panels of D5, E5 and F5). **B and C.** Red arrows indicate regions of TF staining distant from the vessel basement membrane (BM). **D.** Asterisks label the same vessel, black arrows in the crop panel of D5 indicate examples of cells containing punctate structures staining strongly for TF. **E.** Black arrows indicate examples of cells staining intensively for TF throughout the cells, in regions where cells stained intensively for eosin in the H&E stain (black arrowhead). **F.** An orange arrow in the crop panel of F5 indicates an example of the endothelium of a small vessel stained positively for TF. **G.** Purple arrows indicate examples in a region where multiple small vessels contained unidentified structures stained positively for TF. **H.** The overall distribution of vessel phenotypes for TF staining across 8 BU patients. The distribution of fibrin scores associated with each phenotype are represented as pie charts. Increasing fibrin scores corresponding to increasing extension of fibrin staining are represented by stronger colour (0; no fibrin staining with 20 μm, 1–3 are fibrin staining in a <20, 20–30 and >30 μm radius, respectively). The total number of the vessels analysed is shown.

As TFPI expression was depleted by mycolactone *in vitro*, we next considered whether there was evidence for involvement of the extrinsic coagulation cascade in the fibrin deposited in BU patient tissues *in vivo* by examining the localisation of TF staining. In the skin of healthy individuals, TF staining is normally found only in the epidermis and the adventitia of larger (but not smaller) blood vessels, as expected (Figs 4A1-2 and S4). This highly restricted expression pattern is vital to maintain intravascular fluidity, due to the ability of TF to rapidly catalyse activation of the coagulation cascade by binding Factor VII/VIIa. In contrast, TF staining in BU patients was not confined to the sub-endothelium and was instead seen within the tissues in a complex, highly variable pattern between patients.

Abnormal TF staining fell into one of four main categories. First, staining outside of the vessel basement membrane (BM) of larger vessels and instead within the skin tissue stroma (Fig 4B5 and 4C5, red arrows). Second, in presumptive infiltrating cells, either demonstrating granular intracellular staining (Fig 4D5) or intensively throughout each cell (Fig 4E5, black arrows). This pattern of staining was seen in regions that appeared to contain eosinophils based on H&E staining (Fig 4E3, black arrowhead). Third, TF staining was found within the endothelial cells lining smaller vessels (Fig 4F5, orange arrow), although such cells do not normally express TF. Lastly, some vessels contained some structures within the vessel lumen that could not be identified, yet stained strongly for TF (Fig 4G5, purple arrows). Approximately one quarter of vessels with this phenotype were in infiltrated regions (Fig 4G3).

With respect to TF-positive presumptive-infiltrating cells, these were observed within tissue rather than close to vessels. Granular or intensive intracellular TF staining was seen in 5 of the 8 BU patient biopsies, located between 2.2–120.9 μm away from the vessel BM. In four of these patients, it occurred in areas displaying fibrin deposition (compare Fig 4D4 and 4D5).

Vessels were tracked and classified according to their TF expression patterns. Of 830 trackable vessels, 18.2% showed normal TF expression in the adventitia, 45 (5.4%) showed TF staining outside of the vessel BM, 63.3% displayed abnormal TF staining in the vessel endothelium, and 13.1% showed TF within the vessel lumen (Fig 4H). All abnormal expression patterns were associated with an increased chance of having the highest fibrin score. While this was not significantly different for vessels where TF staining was seen in the lumen (chi^2^ vs normal staining pattern *P* = 0.5901), it was distinct in vessels where the endothelium was TF^+^ (chi^2^ vs normal staining pattern *P* = 0.0014). Most strikingly, whilst being relatively rare, vessels that displayed TF staining outside of BM were significantly more likely to have fibrin deposited in the surrounding tissue, (chi^2^ vs normal staining pattern *P* < 0.0001, Fig 4H) and more likely to have fibrin that extended further from the vessel (82.22% exhibiting the highest fibrin score). Notably the coincidence of fibrin and TF outside the BM was often found where there were local signs of tissue necrosis by H&E staining (e.g. Fig 4C3).

In conclusion, this data provides the tantalising possibility that the presence of TF within tissues can trigger fibrin disposition, if the necessary other components of the coagulation cascade, including blood-borne factor VII/VIIa, are also present. It is also tempting to suggest that in these circumstances, the fibrin deposited might trigger local tissue necrosis, presumably due to ischaemia.

### Tissue factor ingress into the stroma surrounding vessels correlates with fibrin deposition

**Fig 5:**
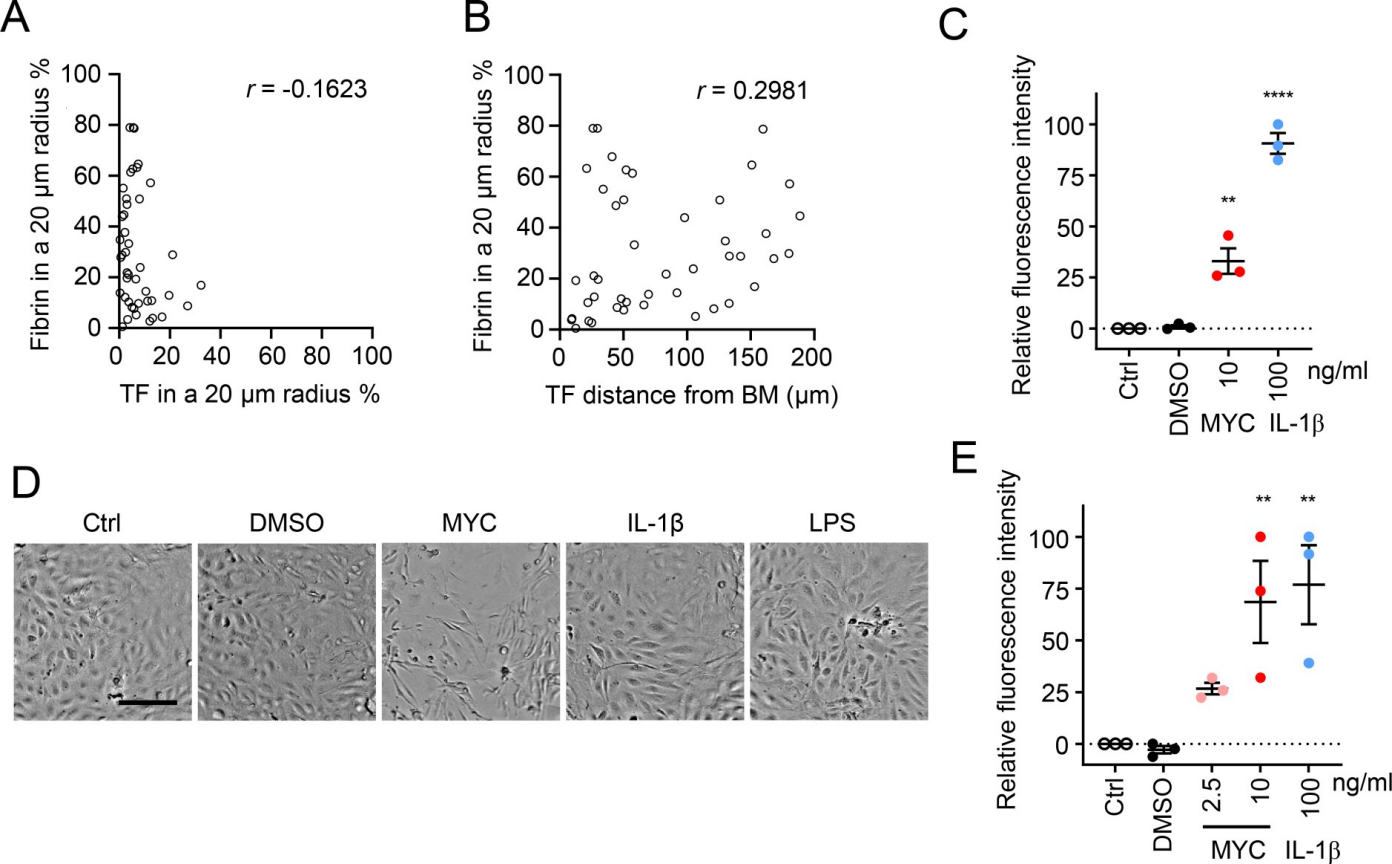
Extravascular tissue factor (TF) is the primary driver of fibrin deposition in BU lesions, potentially driven by an increase in vascular permeability. **A and B.** Correlation between the abundance of fibrin within a 20 μm radius of each vessel and the abundance of tissue factor (TF) in the same radius (**A**) or the distance the TF^+^ signal extended from the vessel basement membrane (BM) (**B**) for 45 trackable vessels. *r* is Spearman’s rank correlation coefficient for each data set. **C-E.** Endothelial cells were exposed to 10 ng/mL mycolactone (MYC), 100 ng/mL IL-1β, 400 ng/mL LPS, 0.02% DMSO or remained untreated for the indicated times then subjected to further analysis. **C.** Permeability of HDMEC on inserts with 0.4 μm pores that received various treatment for 24 hrs. Fluorescence intensity of FITC-dextran in the receiver wells was measured and presented as a % where 100% is the value obtained from transwell lacking a cell monolayer, and 0% is untreated control wells. Values represent the mean of three independent experiments ± SEM. **, *P* < 0.01; ****, *P* < 0.0001. **D.** Endpoint brightfield images of HDMECs exposed to different stimuli and monitored by live-cell imaging for 24 hrs (three independent experiments). Scale bar = 200 μm. **E.** Permeability of HDLEC monolayers, on inserts with 1.0 μm pores that received various treatment mentioned above or 2.5 ng/mL mycolactone (MYC) for 24 hrs. Fluorescence intensity of FITC-dextran in the receiver wells was measured and presented as a % where 100% is the value obtained from transwell lacking a cell monolayer, and 0% is untreated control wells. Values represent the mean of three independent experiments ± SEM. **, *P* < 0.01.

Taken together, the data from BU punch biopsies suggests that the expression of haemostatic markers on the vessel endothelium is not a reliable predictor of fibrin deposition close to that vessel. Instead, fibrin deposition in the tissue seems to arise locally. It is conceivable that activation of the coagulation pathway to generate the thrombin that converts fibrinogen to fibrin, could be initiated by the TF detected within the stroma. There is some evidence to support this in the current analysis. There was no correlation between the percentage of the area positively stained for fibrin and TF within a 20 μm radius of the vessel (*r* = -0.1623, *P* = 0.2869) (Fig 5A). However, there was a moderately positive correlation (*r* = 0.2981, *P* = 0.0467) between fibrin coverage and the distance of TF^+^ signal extended from the same vessel BM (Fig 5B). This suggests that the further the TF has invaded the tissue beyond the stroma, the more likely it is that there will be a high degree of fibrin deposited.

### Mycolactone-dependent vascular permeability as a potential driver of coagulopathy in Buruli ulcer lesions

The generation of fibrin within tissue, a substantial distance away from vessels, after extrinsic coagulation pathway activation by TF would require large plasma glycoproteins to leave blood vessels and gain access to the stromal compartment. Coagulation factors are approximately 50–70 kDa in mass, while cofactors and fibrinogen are larger (~330 kDa). We therefore asked whether mycolactone might induce changes in endothelial cells *in vitro* that might explain the breakdown of vessel integrity and increase of vascular permeability *in vivo*.

Hence, we evaluated the effect of mycolactone on the permeability of HDMEC monolayers to 70 kDa FITC-labelled dextran in a transwell system. Remarkably, exposure to 10 ng/mL mycolactone for 24 hours increased the permeability of the monolayer to 33.1±6.2% of the value seen when cells were completely absent from the transwell (Fig 5C), equivalent to around one-third of that seen after exposure to the proinflammatory cytokine interleukin-1β (IL-1β), a known inducer of vascular permeability [49]. To understand what might be driving this increase, we used live-cell imaging to monitor changes in HDMEC morphology after exposure to mycolactone in comparison to permeability inducing inflammatory stimuli lipopolysaccharide (LPS) and IL-1β (Fig 5D and S1–S5 Videos). Mycolactone-treated endothelial cells initially maintained a normal, cobblestone-like appearance with a few rounded cells undergoing cell division. By 8 hours exposure, cells had developed a distinctive, elongated appearance, which became more evident at 16 hours and predominant at 24 hours (Fig 5D and S3 Video). These changes all preceded the cells succumbing to mycolactone-induced apoptosis ([42] and S3C Fig).

We also wondered whether a mycolactone-dependent increase in permeability might extend to the lymphatic system since BU can sometimes have an oedematous presentation. In human dermal lymphatic endothelial cells (HDLECs), a subpopulation of endothelial cells that actively participate in fluid balance and transport [50,51]. HDLECs and HDMECs had comparable IC_50_ to mycolactone (around 0.67 ng/mL, S3B Fig), but the permeability response of lymphatic endothelial cells to mycolactone was more pronounced in HDLECs. Exposure to 10 ng/mL mycolactone for 24 hours increased the permeability of the monolayer to 68.6±19.8% of the value seen in empty wells (Fig 5E). In the same time window, even 2.5 ng/mL mycolactone could increase Dextran diffusion but this was not statistically significant (26.7±2.8%, Fig 5E).

### Mycolactone-dependent reduction in junctional proteins likely contributes to vascular permeability

The endothelial monolayer is maintained by a diverse system of adhesion proteins, intracellular interacting factors and signalling molecules. Both adherens junctions (mediated by VE-cadherin and catenins) and tight junctions (mediated by Claudin, Occludin and the junctional adhesion molecules (JAMs)) play an important role in maintaining junctional integrity required to prevent large molecules from diffusing across the endothelial cell monolayer [52]. In addition, the angiopoietin-1/ tyrosine kinase with Ig-like loops and epidermal growth factor homology domains (TIE) receptor system controls vascular remodelling and vessel leakage during inflammation [52,53]. VE-cadherin, JAMs, TIE-1 and TIE-2 (or TEK) can all be predicted to be sensitive to the Sec61-dependent effects of mycolactone as they are type I transmembrane proteins. We therefore tested this *in vitro* using primary endothelial cells, comparing the effects to LPS and IL-1β [49,54] since inflammatory conditions often potentiate redistribution of junctional proteins [55] and suppress the expression of TIE-1 [53,56].

**Fig 6:**
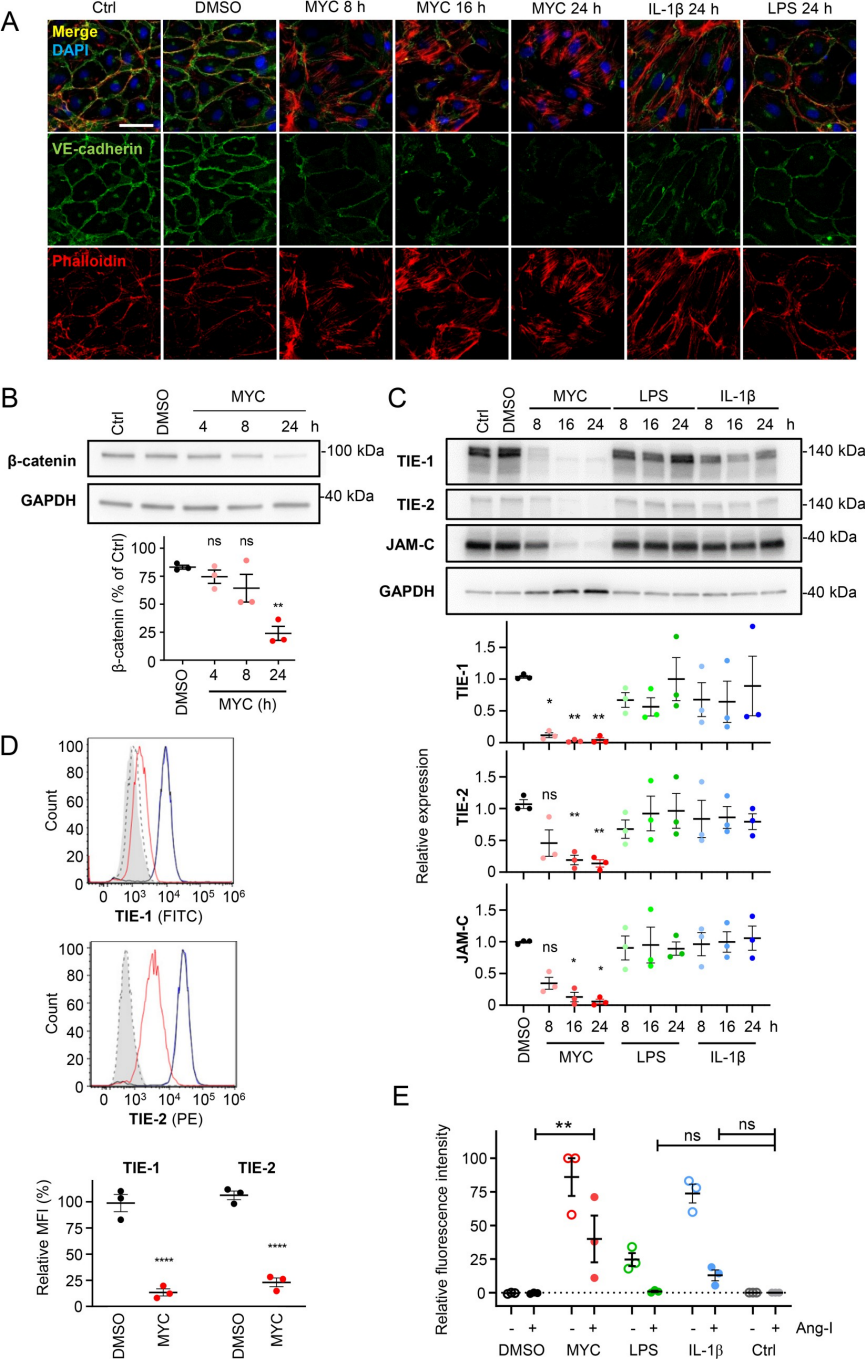
Endothelial junctional proteins are depleted by mycolactone. Endothelial cells were exposed to 10 ng/mL mycolactone (MYC), 100 ng/mL IL-1β or 400 ng/mL LPS for the indicated times then subjected to further analysis. **A.** Treated HDMECs were fixed, immunostained with anti-VE cadherin antibody (green), permeabilised and labelled with TRITC-conjugated phalloidin (red). Nuclei were stained with DAPI (blue). Images are representative of 3 independent experiments. Scale bar = 50 μm. **B.** Treated HDMECs were lysed and subjected to immunoblotting. Immunoblot intensity of β-catenin was normalised according to GAPDH and expressed relative to untreated controls. Values represent the mean of three independent experiments ± SEM. ns, not significant; **, *P* < 0.01. **C.** Treated HUVECs were lysed and subjected to immunoblotting. Immunoblot intensity of TIE-1, TIE-2 and JAM-C was normalised according to GAPDH and expressed relative to untreated controls. Values represent the mean of three independent experiments ± SEM. ns, not significant; *, *P* < 0.05; **, *P* < 0.01. **D**. Treated HDMECs were harvested for flow cytometry analysis of surface proteins, gating on single cells. Histogram plots of FITC (TIE-1) and PE (TIE-2): filled grey, untreated unstained control; dashed black line, isotype control of untreated cells; black, blue and red lines, cells stained with anti-TIE-1 or anti-TIE-2 antibodies, unexposed (black), exposed to DMSO (blue) or MYC (red). Mean fluorescence intensity (MFI) is presented as a % of untreated control (mean ± SEM of 3 independent experiments). ****, *P* < 0.0001. **E.** Permeability of HUVEC monolayers on inserts with 1.0 μm pores that remained untreated (Ctrl) or were exposed to various stimuli for 16 hrs and another 8 hrs with or without 400 ng/mL angiopoietin-1 (Ang-I). Fluorescence intensity of FITC-dextran in the receiver wells was measured and presented as a % where 100% is the value obtained from transwells lacking a cell monolayer, and 0% is untreated control wells. Values represent the mean of three independent experiments ± SEM. ns, not significant; **, *P* < 0.01.

First, we examined the maintenance of adherens junctions by monitoring the expression and localisation of VE-cadherin. VE-cadherin was located along cell-cell junctions and the perinucleus in untreated and control HDMECs (Fig 6A), frequently overlapping with actin, as expected when adherens junctions are intact [57,58]. Following 24 hours exposure to LPS or IL-1β, junctional VE-cadherin was reduced and the junctions appeared discontinuous but perinuclear staining remained comparable. On the other hand, VE-cadherin was severely reduced in mycolactone-exposed cells by 24 hours, with lower levels of expression already apparent at 8 hours. This was also accompanied by a strong reduction in perinuclear staining (Fig 6A). Mycolactone promoted condensation of actin filaments at the endothelial cell edge within 8 hours, as previously observed in epithelial cells [59]. We also quantified expression of the VE-cadherin cytosolic binding partner, β-catenin (CTNNB1), an endothelial barrier modulator that links VE-cadherin to cytoskeleton [60]. CTNNB1 was also significantly downregulated by mycolactone at 24 hours in HDMECs (Fig 6B). Furthermore, the tight junctional protein JAM-C was also depleted by mycolactone, with a significant loss seen at 16 hours (Fig 6C). In contrast neither LPS nor IL-1β caused any significant change in JAM-C expression.

We then investigated the effect of mycolactone on TIE-1 and TIE-2 expression. While exposure of HUVECS to LPS or IL-1β caused small, non-significant changes in total TIE-1 and TIE-2 levels (Fig 6C), mycolactone had a profound effect on both total and cell surface TIE-1 expression in both HUVECS (Fig 6C) and HDMECs (Figs 6D and S5), with an ~80% reduction at 8 hours that further decreased over time. Likewise, mycolactone reduced total TIE-2 abundance after 16 hours in HUVECs (~80% reduction compared to DMSO control, Fig 6C). In HDMECS this decrease did not reach statistical significance (S5 Fig) but cell surface expression was significantly reduced (Fig 6D).

Angiopoietin-1 (ANGPT-1, Ang-1) restores endothelial monolayer integrity via interaction with TIE-2. We therefore assessed the ability of angiopoietin-1 to reverse the vascular permeability induced by mycolactone. In the presence of LPS and IL-1β, inclusion of angiopoietin-1 during the final 8 hours of the 24 hour experiment led to a non-significant (1.0±0.6% and 12.9±4.0%, respectively, Fig 6E) increase in Dextran diffusion compared to the baseline, demonstrating the expected angiopoietin-1-dependent rescue of monolayer integrity. On the other hand, mycolactone-induced permeability was blunted by angiopoietin-1 but remained significantly above the baseline at 40.0±17.4% of the maximum (no cells; Fig 6E).

In conclusion, mycolactone potentiates hyperpermeability of vascular endothelial cell monolayers prior to cytotoxicity through a mechanism distinct from those induced by classic inflammatory stimuli, by targeting expression of multiple key junctional proteins. These findings are in line with mycolactone’s predicted ability to prevent translocation of type I transmembrane proteins VE-cadherin, TIE-1, TIE-2 and JAM-C into the ER. As β-catenin is not a Sec61-substrate, we propose that its depletion is an indirect consequence of VE-cadherin loss, since free β-catenin is destroyed by the phosphodestruction complex [61,62].

### IL-1β aggravates mycolactone-driven endothelial dysfunction

**Fig 7:**
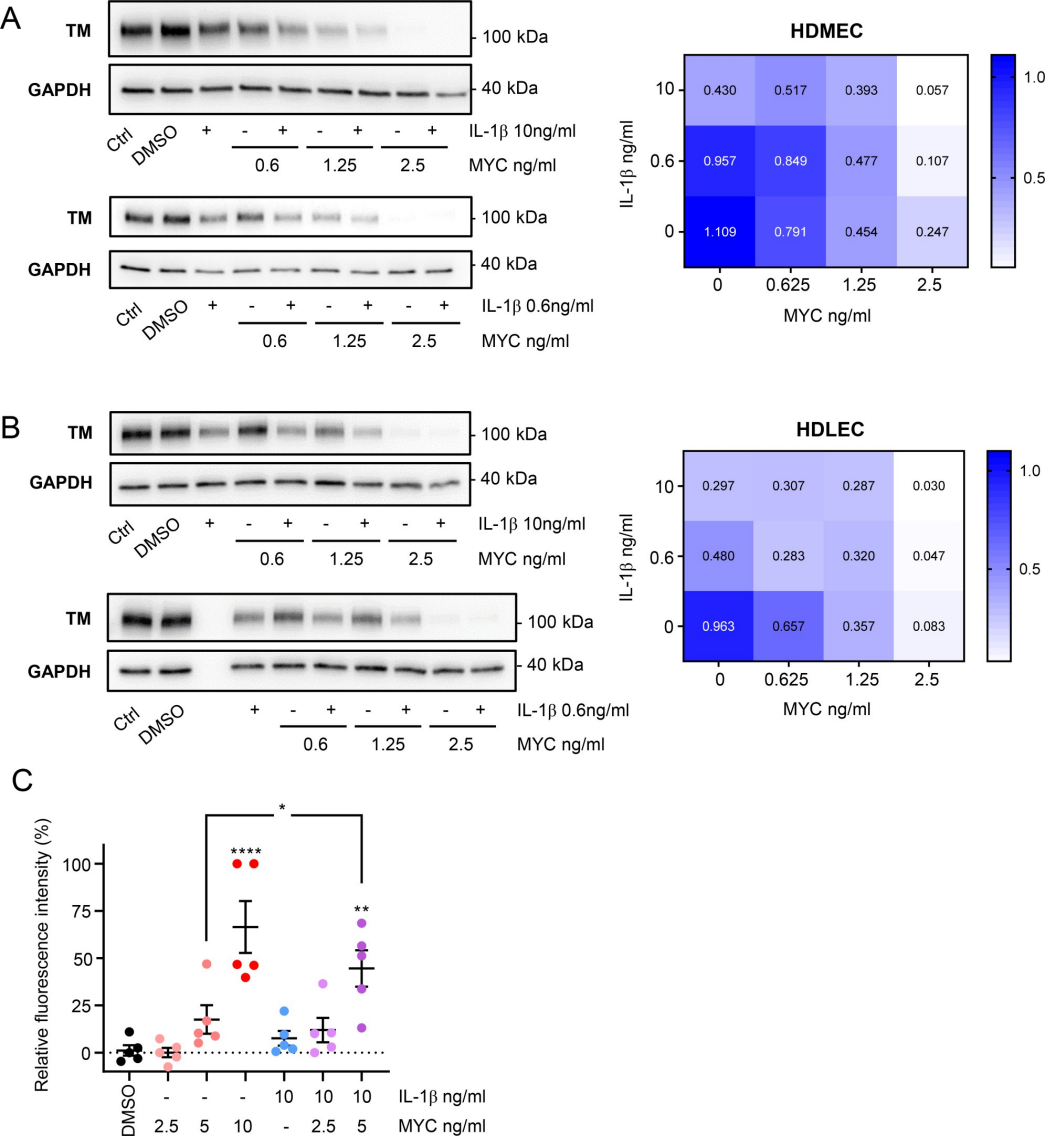
IL-1β aggravates mycolactone-driven endothelial dysfunction. HDMECs (**A**) or HDLECs (**B**) were exposed to different concentrations of mycolactone (MYC), and/or IL-1β for 24 hours, then lysed and subjected to immunoblotting. TM immunoblot intensity was normalised according to GAPDH and expressed relative to untreated controls. Mean expression level from 3 independent experiments is presented as a heatmap where increased TM loss is represented by a paler colour. **C**. Permeability of HDMEC monolayers on inserts with 1 μm pores was quantified after exposure to mycolactone (MYC) and/or IL-1β for 24 hours. Fluorescence intensity of FITC-dextran in the receiver wells was measured and presented as a % where 100% is the value obtained from transwell lacking a cell monolayer, and 0% is untreated control wells. Values represent the mean of five independent experiments ± SEM. *, *P* < 0.05; **, *P* < 0.01; ****, *P* < 0.0001.

Recent work showed that IL-1β can be induced by mycolactone-containing microvesicles and is found in *M*. *ulcerans*-infected tissues [63]. Since IL-1β has been long-known to repress thrombomodulin expression in endothelial cells by a transcriptional mechanism [64], we wondered whether the presence of this alarm cytokine might exacerbate the effect of mycolactone on the microvasculature. First, we addressed whether mycolactone’s ability to supress thrombomodulin expression in primary endothelial cells might be influenced by IL-1β, exposing cells to a range of concentrations of mycolactone and/or IL-1β. As expected, exposure to either 10 ng/mL IL-1β or 2.5 ng/mL mycolactone alone for 24 hours led to a significant depletion in thrombomodulin protein level in both HDMECs (Fig 7A) and HDLECs (Fig 7B). Similar to our observations regarding vascular permeability, HDLEC were more sensitive to both stimuli, with >50% reduction in the presence of 0.6 ng/mL IL-1β (*P* = 0.0132) or 1.25 ng/mL mycolactone (*P* = 0.0004) (Fig 7B). Remarkably, this downward trend was more evident when the endothelial cells were co-exposed to non-saturating amounts of both agents (Fig 7A and 7B). For example, in HDMEC, while each stimulus alone resulted in ~55–75% depletion of thrombomodulin expression, it was barely detectable in endothelial cells exposed to both 2.5 ng/mL mycolactone and 10 ng/mL IL-1β. In HDLEC, the combination of 0.625 ng/mL mycolactone and 0.6 ng/mL IL-1β suppressed thrombomodulin expression to the same extent as 10 ng/mL IL-1β. These effects appear to be additive rather than synergistic.

Since the additive effect was particularly evident in HDMECs, we investigated whether it might also influence vascular permeability in these cells. Here, we used a lower dose of IL-1β, which only slightly increased the permeability of monolayer after 24 hours (7.66±3.89% of the value seen in an empty well, Fig 7C). Mycolactone had a dose-dependent effect on vascular permeability as before, and the additive effect of IL-1β was evident when there was 5 ng/mL mycolactone, increasing the permeability significantly from 17.58±7.57% to 44.62±9.65% (*P* = 0.0021 vs. DMSO; *P* = 0.0268 vs. mycolactone alone). This data suggests that even low amounts of IL-1β in infected tissues might have a further negative impact on haemostatic status and endothelial monolayer integrity where trace amounts of mycolactone are present.

## Discussion

Coagulation is often considered as a frontline host defence response to infection due to trapping of invading bacteria clots [65]; however, some pathogens develop strategies to bypass or utilise clot architecture to gain access to host resources. For example, *Streptococci* generate streptokinase, a plasminogen activator mimic, that promotes fibrinolysis and thus has a positive impact on bacterial metastasis [66]. Coagulase-positive *Staphylococci*, on the other hand, produce coagulase, which rapidly depletes fibrinogen from the plasma and blunts the phagocytic activity of neutrophils [67]. Malaria-infected erythrocytes cause the loss of EPCR and thrombomodulin in brain vessels, leading to subsequent fibrin deposition within the microvasculature [68], which impacts the severity of cerebral malaria [69]. In the case of BU, the skin lesions exhibit large-scale coagulative necrosis in the subcutaneous regions, a distinct pathological phenotype, which has been long associated with the biological effects of the exotoxin mycolactone as it diffuses through the infected tissue [70].

Cumulative reports with *in vitro* or *in vivo* assays have addressed how cell death can be potentiated in response to mycolactone, either directly via the activation of caspase-3, caspase-9 [71] or Bcl-2-like protein 11 [72] with resultant apoptosis, and/or via an integrated stress response leading to ATF4 activation [73,74]. These pathways may also involve disruption of actin cytoskeleton [59]. Apoptosis of mycolactone-exposed cells is clearly linked to its effect at the Sec61 translocon. Cells carrying specific mutations in the gene encoding the major subunit, Sec61α, are resistant to mycolactone-driven cytotoxicity and continue to proliferate in its presence [23,73,75]. They are also resistant to mycolactone-induced cellular stress [73,74], autophagy [73,76] and Ca^2+^ leak from the ER [77]. More recently we proposed an indirect mechanism may operate *in vivo*, via mycolactone’s influence on endothelial cell anticoagulant function and resultant tissue ischemia [42]. However, to what extent coagulation is dysregulated in BU has been unclear. This present work demonstrates several abnormal haemostatic phenotypes in the dermal microvasculature of BU patients that includes: 1) loss of key endothelial proteins such as the endothelial adhesion molecule CD31, the anticoagulant thrombomodulin, and the platelet binding partner vWF, and 2) highly abnormal expression patterns of TF that suggest a loss of containment. These changes have the potential to generate the pathogenic fibrin deposition seen in the skin lesions and imply that infection with *M*. *ulcerans* may cause a localised, disturbed haemostatic status. This conclusion is reinforced by *in vitro* data using primary endothelial cells, where mycolactone can profoundly reduce endothelial cell junctional proteins including TIE-1, TIE-2, JAM-C as well as VE-cadherin and β-catenin, and potentiate vascular and lymphatic hyperpermeability. Moreover, mycolactone limits the expression, and secretion of both vWF and TFPI.

Mechanistically, a combination of mycolactone’s action at the Sec61 translocon, and increased local concentration of IL-1β likely explains the endothelial dysfunction seen in BU. The single-pass type I membrane proteins thrombomodulin, CD31, VE-cadherin, JAM-C, TIE-1 and TIE-2 [42], the secretory proteins vWF and GPI-anchored TFPI all require Sec61-dependent co-translational translocation into the ER lumen to complete their biosynthesis. These classes of protein are all inhibited by mycolactone [24,25]. In *in vitro* model systems, the mycolactone concentration is uniform, whereas levels in BU skin lesions may vary considerably. However, where the local mycolactone concentration is high enough, depletion of these molecules will also occur. The range of endothelial marker expression seen across different specimens and sections from the same specimen may therefore reflect local variations in mycolactone concentration. However, while there is evidence for mycolactone within BU lesions [14,78], current methodology lacks the spatial resolution to understand exactly how much mycolactone is present at specific sites.

Despite a shared dependence on Sec61, there was less correlation between endothelial markers than expected in the disordered skin in BU. For instance, while many vessels lacked both thrombomodulin and vWF, some lost expression of one marker but not the other. Since it was recently shown that IL-1β is present in BU skin lesions, also with variable intensity across biopsy samples [63], we tested the hypothesis that IL-1β may add an additional layer of complexity in the *in vivo* setting. We showed that mycolactone and IL-1β have an additive effect *in vitro*, suggesting that the variation in marker expression could be caused by the cumulative effect of local mycolactone and IL-1β. In this context, it is also worth noting that the distance between our serial tissue sections for these markers is 24 μm inclusive. Given the focal nature of the staining for the same markers across the section (see S2A Fig), distance between sections could also be a contributing factor.

These BU patient biopsies showed widespread changes to vWF localisation with only a minority of vessels displaying a normal expression pattern. In healthy tissue, vWF is expressed solely in endothelial cells, contained within WBPs, which also contain a variety of other components regulating inflammation as well as vascular modulators, including angiopoietin-1 [35]. Endothelial cell activation results in WBP exocytosis and the formation of vWF multimeric strings which capture platelets [79]. It is possible that WBP exocytosis explains the pattern we observed, particularly in immune cell infiltrated regions, where vWF was lost from endothelial cells and instead observed in the adjacent intravascular space. This pattern of expression has been previously reported in the dermal vasculature of patients with malignant melanoma [80] as well as alveolar septa and large blood vessels of patients with malaria-associated acute respiratory distress syndrome [46]. In melanoma vasculatures, CD42^+^ platelet aggregates were also seen bound to intravascular vWF fibres [80], due to an endothelial glycocalyx shedding-dependent process [81]. We did not observe platelet aggregates in these vessels in our BU patients, although we cannot completely rule out that it was a technical limitation of the antibody used to detect them [82].

Another haemostatic factor with a grossly abnormal expression pattern was the extrinsic pathway initiator TF, which plays a critical role in both fibrin generation and wound repair [83]. Exposure of TF to blood is sufficient to initiate clotting, due to its cofactor activity in the activation of factors IXa and Xa by factor VIIa [84]. Consequently, TF expression in the skin is normally restricted to the epidermis and the adventitia surrounding larger vessels as a so-called “haemostatic envelope” [84], and its activity is usually tightly regulated by an inhibitor, TFPI [40]. Changes in TF expression have been reported in many inflammatory thrombotic conditions [83,85], as well as in infections such as tuberculosis [86,87]. Myeloid cells do not normally express TF, but during *M*. *tuberculosis* infection, TF expression is observed in macrophages along with conspicuous fibrin deposition within granulomas [87,88]. Mice genetically altered to have low TF expression [86] or TF deficiency in myeloid cells [88] do not show these changes.

Whether the endothelium can be a cellular source of the TF in pathogenic conditions remains highly controversial [83,89]. It is well-established that inflammatory agents such as LPS oxidized low density lipoprotein and IL-1β can induce TF mRNA and protein expression in monocytes and macrophages both *in vitro* and *in vivo* [90–92]. On the other hand, while endothelial cells can be induced to express TF *in vitro* in response to LPS and IL-1β [49,93,94] the evidence that this takes place *in vivo* as part of a pathophysiological process is not so clear. For instance, endothelial-specific TF-knockout mice display little change in pathogenesis in a range of disease models [83,95]. Much evidence supports a view that procoagulant TF always arises via activated mononuclear cells and that the detection of TF in other cells is a consequence of microparticle uptake [83,96]. Indeed, early reports that eosinophils could express TF following activation [97] were more recently shown to be explained by their uptake of monocyte-derived microparticles [89]. While this is a highly complex and also controversial area, many cell types including endothelial cells, platelets, and monocytes/macrophages have all been shown to be capable of producing TF-bearing microparticles *in vitro*, but monocyte-derived microparticles have been shown to be the most thrombogenic [98]. As well as providing the extrinsic clotting pathway initiator TF, microparticles are also thought to promote coagulation by providing a rich negatively charged surface for the amplification of the coagulation cascade at their location [83]. The presence of circulating TF-bearing microparticles has been reported in patients with cancer-associated thrombosis [99], venous thromboembolism [100], cardiac bypass surgery [101], Behҫet’s disease [102] and sepsis [103].

In the BU patients we investigated, epidermal expression of TF was not altered, but within the dermis and subcutis extremely disordered TF expression was observed. In stark contrast to healthy skin, TF was detected in the endothelial cells of >60% of tracked vessels, as well as in infiltrating cells in five patients with either granular, or in one patient with whole-cell, staining. This finding, reminiscent of inflammatory thrombotic conditions, such as sepsis and atherosclerosis [83,85,89], was surprising because of mycolactone’s well-characterised immunosuppressive and anti-inflammatory effects [78]. Once again, IL-1β may play an important role here. We speculate that the TF^+^ endothelial cells and presumptive eosinophils we observed could have arisen by uptake of TF-bearing microparticles induced by IL-1β within the lesions. It is unlikely that TF in the endothelium has much influence over disease progression in these BU patients, as fibrin deposition was little altered around these vessels even when the anticoagulant thrombomodulin was also lost from the endothelial cell surface. On the other hand, we observed, albeit rarely, TF^+^ cells (presumably eosinophils) in regions of BU lesions containing intensive fibrin staining. Notably, eosinophils have also been reported as the cellular source of TF seen in another skin condition, chronic urticaria [104]. It is also possible that some of the infiltrating TF^+^ cells we observed are activated macrophages that have been induced to express TF. *M*. *ulcerans* infection is known to include a phase where macrophages are transiently infected with bacteria [105], and the death of these cells due to the cytotoxic effects of mycolactone could conceivably be another source of microparticles. Whether microparticles of any origins play an important role in BU will be the subject of future investigations.

The strongest association we observed with fibrin deposition was diffuse TF staining extending away from larger vessels, up to 200 μm into tissue. A key question that arose was; how do TF and the other plasma clotting factors required to produce thrombin and consequently process fibrinogen to fibrin access these sites? Using an *in vitro* vascular permeability assay, we showed that mycolactone potentiates the passage of 70 kDa-size dextran through monolayers of HUVECs, HDMECs and HDLECs, and also provided mechanistic data showing that a variety of junctional and vascular permeability modulating proteins are depleted in the presence of mycolactone. This data builds upon previous work showing that CD31 and thrombomodulin, which also act as essential regulators for endothelial tight junctions [106–108], are also downregulated by mycolactone [42]. Hence, a disturbed endothelial physical barrier between plasma and tissue could be an unavoidable phenotype in BU. A leaky vasculature is well-established to result in increased immune cell invasion, as well as allowing plasma components and fluids to enter tissue [109,110]. Theoretically, the disruption of the barrier between blood and tissue could initiate a clotting cascade as blood-borne clotting factors engage with their sub-endothelium located binding partners or activators such as TF [111]. However, further evidence is needed from animal models before such a process can be certain.

Pro-inflammatory cytokines and pathogen-derived molecules are known to affect both microvascular and lymphatic endothelial barriers and augment vascular permeability [56,109,112], and this can cause tissue oedema in many disease conditions [32,105,108]. Many pathogens also target cell-cell junctions in order to cross the barrier and colonise in tissues. Interestingly, *Mycobacterium marinum*, a close genetic relative of *M*. *ulcerans*, targets vascular integrity to aid its multiplication in granulomas [106]. In BU, oedematous forms are seen in around 5% of patients [113]. Further work will be required to establish how early in the infection process vascular permeability increases, and to understand whether it has a direct impact on *M*. *ulcerans* growth. In the present study, mycolactone demonstrated a broad and rapid effect, limiting the biosynthesis of endothelial cell junctional proteins, triggering morphological changes within 16 hours and disrupting monolayer integrity. Mycolactone’s effect on vascular permeability *in vitro* occurred much earlier (within 24 hours) than its cytotoxic effects (more than 72 hours in HDMECs [42] and HUVECs) and at an extremely low dose (10 ng/mL), even lower in the presence of small amounts of IL-1β. Our *in vitro* data also suggest that pharmacological interventions targeting angiopoietin-1/ TIE receptor system [114] would probably offer limited protection to vasculatures where *M*. *ulcerans* or mycolactone is present.

Taken together, both our cell-based studies and histopathological analysis in patient specimens illustrate a hypercoagulative microenvironment that develops during BU disease progression. It seems likely that the fibrin deposition in BU lesions is not driven by platelet aggregation; instead, it may be a consequence of factor VIIa engaging with sub-endothelial TF following disturbance of endothelial monolayer integrity and exacerbated by loss of its natural inhibitor TFPI. While the current study provides more evidence supporting the ‘indirect’ mechanism of mycolactone-induced necrosis, more work is needed. We have not yet analysed any potential contribution of the intrinsic clotting pathway; therefore, it cannot be excluded. A correct balance of TF-driven coagulation and subsequent fibrin formation is critical in wound healing [115]. Hence, application of anticoagulants along with antibiotics could help neutralise the pro-thrombotic phenotype seen in BU patient skin lesions, and ultimately improve healing rate. Of note, complementary anticoagulant heparin alongside standard anti-tubercular antibiotics treatment has been previously employed to treat a case of facial BU. In this patient, facial oedema was reversed by heparin intravenous injection [116], suggesting lowering haemostatic status may attenuate BU disease progression.

## Materials and methods

### Ethics statement

Ethical approval for analysing BU patient punch biopsies was obtained from the Ethikkommission beider Basel, Basel, Switzerland and the provisional national ethical review board of the Ministry of Health Benin (N IRB00006860) as well as from the Cameroon National Ethics Committee and the Ethics Committee of the Heidelberg University Hospital, Germany (ISRCTN72102977). A favourable ethical opinion for analysing normal human skin was given by the Faculty of Health and Medical Science Ethics Committee of the University of Surrey (1174-FHMS-16). The normal human skin samples were collected by the Whiteley Clinic, Guildford, Surrey or were purchased from AMS Biotechnology. Written informed consent was obtained from adult patients or the guardians of child patients. The research related to human tissues complies with the ethical processes of University of Surrey.

### Histological analysis of human skin samples

Buruli ulcer punch biopsies (4 mm) were collected previously [117] and reanalysed for the current study. The biopsies from 4 male and 4 female patients included lesions on both the upper and lower extremities, from all WHO lesion categories [1]. Clinical features of these patients are summarised in Table 1. In BU patients displaying ulcerated lesions, punch biopsies were taken 1 cm inside the outer margin of the induration surrounding the ulcer. Otherwise, punch biopsies were collected from the non-ulcerated centre of the skin lesions. After removal, tissues were fixed in 10% neutral buffered formalin, transported, embedded in paraffin and sectioned. Five normal human skin tissue blocks were made from 4 mm punch biopsies collected by The Whiteley Clinic or surgical removal specimens (< 1cm^2^) collected by AMS Biotechnology-collaborated research clinical centres.

Nine serial tissue sections of each patient were (immuno-)histochemically stained in the following order: haematoxylin-eosin, fibrin, thrombomodulin (CD141), CD61, CD31 (Platelet endothelial cell adhesion molecule; PECAM-1), SMA (perivascular cell marker), Ziehl-Neelsen, TF and vWF. For immunohistochemical staining, 5-μm skin tissue sections were deparaffinised, endogenous peroxidase quenched, epitope unmasked (either in preheated pH 6 citrate buffer or treated with proteinase K, DAKO) and blocked with normal horse serum (Vector Laboratories). The tissue sections were incubated with primary antibody overnight at 4°C and biotin-conjugated horse anti-mouse/ rabbit IgG (Vector Laboratories) for 30 minutes at room temperature. Primary antibodies were as follows: CD31 (M0823, DAKO), thrombomodulin (M0617, DAKO), SMA (NCL-SMA, Novocastra), CD61 (M0753, DAKO), vWF (ab6994, Abcam), TF (TF218; generous gift from Professor Wolfram Ruf, Scripps Research Institute) and Fibrin (59D8 [118]; generous gift from Professor Charles Esmon, Oklahoma Medical Research Foundation). Staining was performed using VECTASTAIN Elite ABC kit and Vector NovaRED peroxidase substrate (Vector Laboratories). Counterstaining was performed with Shandon Harris Haematoxylin (Thermo Fisher Scientific). Anti-TF and vWF antibodies and their respective matched isotype controls were introduced to healthy skin tissue slides to rule out unspecific signals. High resolution images of all slides were scanned using either an Aperio slide scanner (Leica Biosystems), Panoramic digital slide scanners (3DHISTECH) or the Hamamatsu slide photometry system (Hamamatsu Photonics). Scanned images were further analysed using ImageScope software (Leica Biosystems) or CaseViewer (3DHISTECH). In some cases, photographs were taken with Micropix microscope camera (acquisition software Cytocam) attached to a Yenway CX40 laboratory microscope (Micropix).

### Vessel identification and marker analysis

In the current work, we did not analyse the highly necrotic regions of the BU punch biopsy samples. Our analysis has focused exclusively on adjacent regions of the biopsies that had no submacroscopic appearance of coagulative necrosis present in the corresponding H&E section, as well as plentiful non-necrotic blood vessels based upon positive staining for SMA or CD31. These were defined independently by a pathologist prior to the start of analysis. Two patient biopsies showed two distinct regions that contained plentiful non-necrotic blood vessels, therefore a total of ten representative areas were analysed for each biomarker. Once the regions were chosen, all blood vessels within them were analysed, regardless of whether nearby tissue displayed microscopic signs of necrosis, in order to facilitate an unbiased analysis. Throughout this manuscript, we refer to these as “least-necrotic” regions. To determine the proportion of the total biopsies that was analysed, the pixel areas were measured using NIS Elements Basic Research (Nikon, Tokyo, Japan) (version 5.21.03).

Tracked vessels were initially identified by morphology and positive staining for SMA using the region of interest (ROI) tool on NIS Elements Basic Research (Nikon, Tokyo, Japan) (version 4.6). This allowed for the same vessels to be easily identified in the other sections. Structures with vessel morphology that stained positively for the known endothelial markers CD31, thrombomodulin, or vWF in their corresponding sections were also identified and then tracked in the corresponding sections. Sweat glands stain for SMA, but do not have endothelium and do not stain positive for CD31, thrombomodulin or vWF [119,120], therefore the morphology of singly SMA^+^ structures was carefully considered before assigning them as vessels. Fibrin deposition and TF expression pattern around these vessels, if identifiable, was categorised. Note that not every vessel could be identified within all sections, therefore the number of vessels analysed always represents the number that could be identified through all the relevant sections, and thus varies from analysis to analysis.

To analyse fibrin deposition that was largely seen within the tissues, two different approaches were taken. First, the distance that fibrin staining extended from the basement membrane was categorised based on measurements taken with Aperio ImageScope (version 12.3.3). Vessels with no fibrin staining within 20 μm were scored as 0. Fibrin-positive vessels received increasing scores the further the staining extended (1; <20 μm radius, 2; 20–30 μm radius, 3; >30 μm radius). Second, fibrin staining around each vessel was quantified using the calibrated ruler tool of NIS Elements Basic Research (version 4.6 and 5.21.01). Here, the threshold for positive staining was first defined within random images, which was then applied to all vessels. Then, a 20 μm area around all vessels was measured and traced again with the ROI tool. The area of each original vessel was subtracted from the area of its 20 μm radius in order to give the area in which fibrin staining was to be quantified. Any staining within this was then calculated as a percentage of the 20 μm area around each vessel.

To analyse the unusual TF staining pattern observed, TF staining coverage within 20 μm was quantified as above for fibrin using the calibrated ruler tool of NIS Elements Basic Research (version 5.21.01), and is expressed as percentage of the area that had staining intensity above the positivity threshold. In addition, the distance that the TF signal extended from the individual vessel’s basement membrane was measured by CaseViewer (version 2.2).

### Reagents

Synthetic mycolactone A/B (generous gift of Professor Yoshito Kishi, Harvard University) [121] and its solvent control dimethyl sulfoxide (DMSO, Sigma) were used in cell-based studies. Human recombinant IL-1β was from Gibco. Angiopoietin-1 was from R&D Systems. Lipopolysaccharide (LPS) from *E*. *coli*, serotype O55:B5 (TLR-grade) was from Enzo Life Sciences.

### Cell culture and treatment

Primary human dermal microvascular endothelial cells (HDMEC; LONZA), human umbilical vein endothelial cells (HUVECs, PromoCell) and human dermal lymphatic endothelial cells (HDLEC; PromoCell) from two donors were used and cultured according to manufacturer’s recommendations in Endothelial cell growth medium MV2 (PromoCell). Subconfluent cells were treated with either 10 ng/mL mycolactone, DMSO equivalent to mycolactone dose, 100 ng/mL IL-1β or 400 ng/mL LPS for 24 hrs or as indicated in figure legends. For real-time imaging, endothelial cells were plated onto 24-well plates overnight, treated as indicated and imaged every 30 minutes by zenCELL Owl incubator microscope (LabLogic) for 24 hrs. Time lapse videos were generated with zencell-owl software (version 3.3, innoME GmbH).

### Vascular permeability assay

Endothelial cells were seeded on cell culture inserts containing 0.4 (Millipore) or 1 μm pores (Falcon) with a polyethylene terephthalate membrane. Cells were treated as indicated, in both insert and receiver wells. After 24 hrs, fluorescein isothiocyanate (FITC)-conjugated dextran (70 kDa, Millipore) was applied to each insert for 20 minutes. The fluorescence intensity of the solution in the lower chambers was then assessed by a fluorescent plate reader (FLUOstar Omega, BMG Labtech) with excitation/ emission wavelength at 485/ 530 nm. Fluorescence intensity was normalised to untreated control wells with an intact monolayer of endothelial cells (minimum) and expressed as a percentage of subtracted value obtained from wells where the insert had no cells (maximum). In some experiments, 400 ng/mL angiopoietin-1 was added into both the insert and receiver wells 16 hrs after the initial treatment. Its effect was then evaluated after 8 hrs with the same procedure described above.

### Cell viability assay

Endothelial cells (4 x 10^3^ cells) were seeded onto 96-well plates and treated with a variety of doses of mycolactone the following day. Serial dilutions of mycolactone from 50 to 0.098 ng/mL or solvent control equivalent to highest mycolactone dose (0.1% DMSO) was applied to the cells; on the fifth day viability was assayed using alamarBlue Cell Viability Reagent (Invitrogen) with excitation/ emission wavelength at 560/ 590 nm by a plate reader (FLUOstar Omega, BMG Labtech). Values were normalised to the control wells treated with DMSO and are presented as survival rate (%). Alternatively, HUVECs exposed to 10 ng/mL mycolactone, 0.02% DMSO, 400 ng/mL LPS or 100 ng/mL IL-1β for 24 hours were assayed with CellEvent Caspase-3/7 green detection reagent (Invitrogen) as described previously [73].

### Immunochemical analysis

Western blot analysis was carried out using standard techniques, following cell lysis with 1X RIPA buffer (Sigma) supplied with proteinase inhibitor cocktail (Sigma), separation by electrophoresis on 10% polyacrylamide gels and transfer to PVDF membranes (ThermoFisher Scientific). Antibodies used were thrombomodulin/TM (sc-13164, Santa Cruz), TFPI (AF2974, R&D Systems), TIE-1 (AF619, R&D Systems), TIE-2 (AF313, R&D Systems), JAM-C (AF1189, R&D Systems), β-catenin (sc-7963, Santa Cruz Biotechnology) and GAPDH (AM4300, Ambion). Immunofluorescence staining was performed on paraformaldehyde-fixed cells. To visualise intracellular granules or actin cytoskeleton, cells were permeabilised with 0.5% Triton X-100 for 2 minutes or 0.25% NP-40 for 5 minutes, respectively. Antibodies used were vWF (ab6994, abcam), VE-cadherin (D87F2, Cell Signaling), TRITC-conjugated phalloidin (FAK100, Sigma-Aldrich) and Alexa Fluor 488 goat anti-rabbit IgG (H+L) (ThermoFisher Scientific). Nuclei were visualised with DAPI. Images were acquired with Nikon A1M confocal laser scanning unit attached to an Eclipse Ti-E microscope.

To assess the TFPI protein level in conditioned medium, an in-house ELISA was performed. In brief, samples or TFPI recombinant protein standards (ranging from 1000 to 8 ng/mL, 2974-PI. R&D Systems) were coated onto immunoplates (MaxiSorp, Nunc). Following overnight incubation, individual wells were blocked with 1% bovine serum albumin. Antibodies used were TFPI (AF2974, R&D Systems), HRP Rabbit anti-goat IgG (H+L), and Avidin anti-HRP (Invitrogen). The reaction was developed with 3, 3’, 5, 5’-tetramethylbenzidine substrate (Invitrogen), stopped by 1M H_2_SO_4_ and read at 450 nm by a plate reader (FLUOstar Omega, BMG Labtech).

To determine the effect of mycolactone on the release of vWF from Weibel-Palade bodies, HDMECs were pre-incubated with DMSO or 7.8ng/mL mycolactone for 24 hours, washed twice with serum free medium, then stimulated with 2 U/mL thrombin for 10 minutes. vWF protein levels were then quantified using an in-house vWF ELISA. Anti-human vWF antibody (A0082, Dako) was coated onto immunoplates overnight at 4°C and blocked with 2% BSA in PBS for 1 hour at room temperature. After blocking, the plate was washed three times with wash buffer (0.05% (v/v) Tween-20 in PBS), samples and standards (NIBSC) ranging from 1000 mIU/mL—4 mIU/mL were added to the wells and the plate was incubated at room temperature for 2 hours. Then, vWF was detected with HRP-conjugated rabbit anti-human vWF (P0226, Dako) followed by 3, 3’, 5, 5’-tetramethylbenzidine substrate (Invitrogen), stopped by 1M H_2_SO_4_ and read at 450 nm by a plate reader (FLUOstar Omega, BMG Labtech).

### Statistical analysis

Statistical analysis was carried out using GraphPad Prism version 8 (San Diego, USA). Categorical data was analysed using Chi-square test of association. Yate’s correction for continuity was introduced to TF categorical data set as some observed values were below 5. Fibrin coverage per TM^+^ or TM^-^ vessel was assessed using Mann Whitney *U* non-parametric test. Fibrin coverage versus TF coverage or distance to BM per vessel with TF seen outside of BM was assessed using D’Agostino-Pearson normality test; as data set displayed a non-Gaussian distribution, correlation used the method of Spearman. Data otherwise was accessed using a one-way ANOVA and Dunnett’s multi-comparison test. Unless otherwise indicated, statistical comparison for *in vitro* assays was vs DMSO-treated controls.

## Supporting information

S1 Fig The expression patterns of haemostatic modulators in 8 BU patient skin biopsies. Histological sections from 8 BU patient punch biopsies stained with eosin (H&E) or antibody against fibrin, TM, CD61, CD31, SMA, TF and vWF and counterstained with Haematoxylin. The least-necrotic regions identified by a pathologist are outlined in red; vessels in these areas were tracked and analysed for this study. The regions that were infiltrated with immune cells are indicated in the relevant vWF panel, and are outlined in blue. Scale bar = 1 mm.
(TIF)

S2 Fig General features of BU patient skin biopsies. **A.** Comparison of the staining patterns with the same anti-SMA or anti-fibrin antibody and conditions seen for the same punch biopsies at different positions in the tissue block. The “initial section” displays those performed for Ogbechi et al., 2015 [42], whereas the “contiguous section” was performed for the present work. Arrows in different colours label the same vessel identified in different tissue sections. Scale bar = 500 μm. Note how the vessel phenotype can vary even over small distances. **B.** An example of vessel identification and labelling (in colours with individual number indicated) in serial tissue sections stained with anti-SMA and CD31 antibody. **C.** Mycobacterial clusters (in purple, indicated with arrows) are identified in histological sections from 2 BU patient punch biopsies with Ziehl-Neelsen staining. Scar bars as indicated.
(TIF)

S3 Fig vWF expression in BU patient specimen and in primary HDMECs. **A.** Histological sections of a healthy subject or BU patient stained with anti-vWF antibody, the respective isotype control or secondary antibody alone and counterstained with Haematoxylin. **B.** Cell viability of HDLECs, HDMECs and HUVECs exposed to a variety doses of mycolactone for 5 days was determined using alamarBlue assay and presented as a % where 100% is the value obtained from cells treated with solvent control DMSO. **C.** Viability of HUVECs that were untreated or exposed to 0.02% DMSO, 10 ng/mL mycolactone (MYC), 100 ng/mL IL-1β or 400 ng/mL LPS for 24 hrs using CellEvent detection kit. The number of active caspase 3/7 and PI-positive cells were counted per field and expressed as a % of total number of cells. Three different fields representing the top, bottom and middle part of plate were taken. **D.** HDMECs were treated with 10 ng/mL of mycolactone (MYC), 0.02% DMSO or untreated (Ctrl) for 24 hours. Cells were fixed, permeabilised and immunostained with anti-vWF antibody. vWF-containing granules are shown in green and nuclei stained with DAPI (blue). Scale bar = 20 μm.
(TIF)

S4 Fig Tissue factor (TF) expression patterns in BU patient specimen. Histological sections of a healthy subject or BU patient stained with anti-TF antibody, the respective isotype control or secondary antibody alone and counterstained with Haematoxylin. Scale bar as indicated.
(TIF)

S5 Fig Endothelial TIE1/2 expression in response to mycolactone. HDMECs were exposed to 0.02% DMSO, 10 ng/mL mycolactone (MYC) or remained untreated for the indicated times, lysed, and subjected to immunoblotting. Immunoblot intensity of TIE-1 and TIE-2 was normalised according to GAPDH and expressed relative to untreated control. Values represent the mean of three independent experiments ± SEM. ns, not significant; ****, *P* < 0.0001.
(TIF)

S1 Table Summary of marker analysis per identified vessel in least-necrotic regions. Score (0–3) per analysed marker is indicated in the info sheet. Score of SMA, CD31, TM, fibrin, CD61, vWF and tissue factor that links to respective vessel ID, patient ID (#1–8) and location of least-necrotic regions (D as dermis and S as subcutis) is summarised in the “908 identified vessels” sheet.
(XLSX)

S1 Video Live imaging of untreated endothelial cells. Untreated HDMECs were real-time monitored at 30 min intervals using zenCELL Owl incubator microscope for 24 hours. Time lapse videos were generated with zencell-owl software. Time stamp and scale bar as indicated. (three independent experiments).
(MP4)

S2 Video The effect of DMSO on endothelial cells. HDMECs were exposed to 0.02% DMSO and real-time monitored at 30 min intervals using zenCELL Owl incubator microscope for 24 hours. Time lapse videos were generated with zencell-owl software. Time stamp and scale bar as indicated. (three independent experiments).
(MP4)

S3 Video The effect of mycolactone on endothelial cells. HDMECs were exposed to 10 ng/mL mycolactone and real-time monitored at 30 min intervals using zenCELL Owl incubator microscope for 24 hours. Time lapse videos were generated with zencell-owl software. Time stamp and scale bar as indicated. (three independent experiments).
(MP4)

S4 Video The effect of IL-1β on endothelial cells. HDMECs were exposed to 100 ng/mL IL-1β and real-time monitored at 30 min intervals using zenCELL Owl incubator microscope for 24 hours. Time lapse videos were generated with zencell-owl software. Time stamp and scale bar as indicated. (three independent experiments).
(MP4)

S5 Video The effect of LPS on endothelial cells. HDMECs were exposed to 400 ng/mL LPS and real-time monitored at 30 min intervals using zenCELL Owl incubator microscope for 24 hours. Time lapse videos were generated with zencell-owl software. Time stamp and scale bar as indicated. (three independent experiments).
(MP4)
