# Supplementary figures and images for: Aberrant stromal tissue factor localisation and mycolactone-driven vascular dysfunction, exacerbated by IL-1β, are linked to fibrin formation in Buruli ulcer lesions
Source: PLoS Pathog. 2022 Jan 31;18(1):e1010280. doi: 10.1371/journal.ppat.1010280 (PMC8846541; doi:10.1371/journal.ppat.1010280)

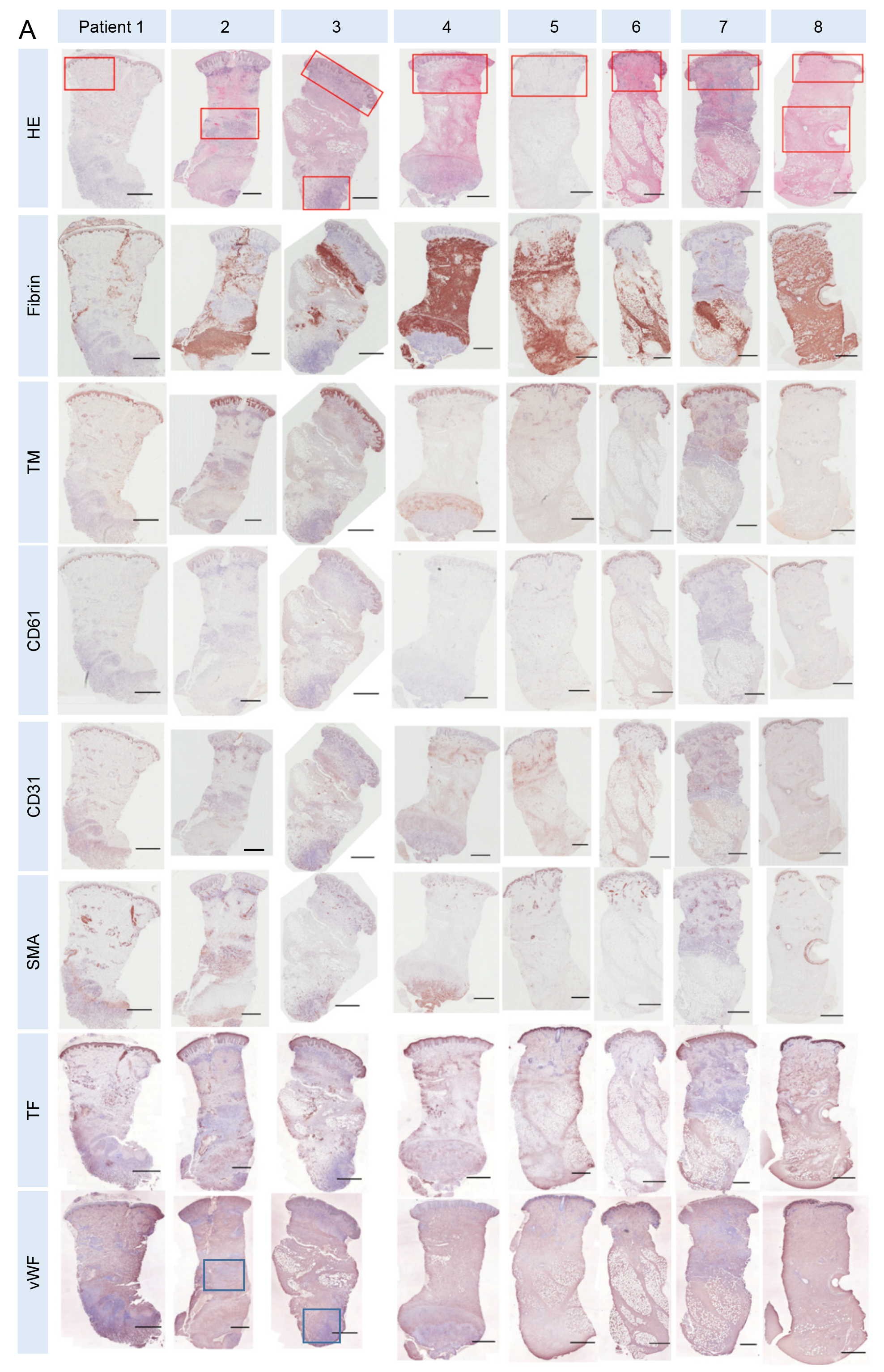

Supplement: S1 Fig — Histological sections from 8 BU patient punch biopsies stained with eosin (H&E) or antibody against fibrin, TM, CD61, CD31, SMA, TF and vWF and counterstained with Haematoxylin. The least-necrotic regions identified by a pathologist are outlined in red; vessels in these areas were tracked and analysed for this study. The regions that were infiltrated with immune cells are indicated in the relevant vWF panel, and are outlined in blue. Scale bar = 1 mm. (TIF) [file ppat.1010280.s001.tif]

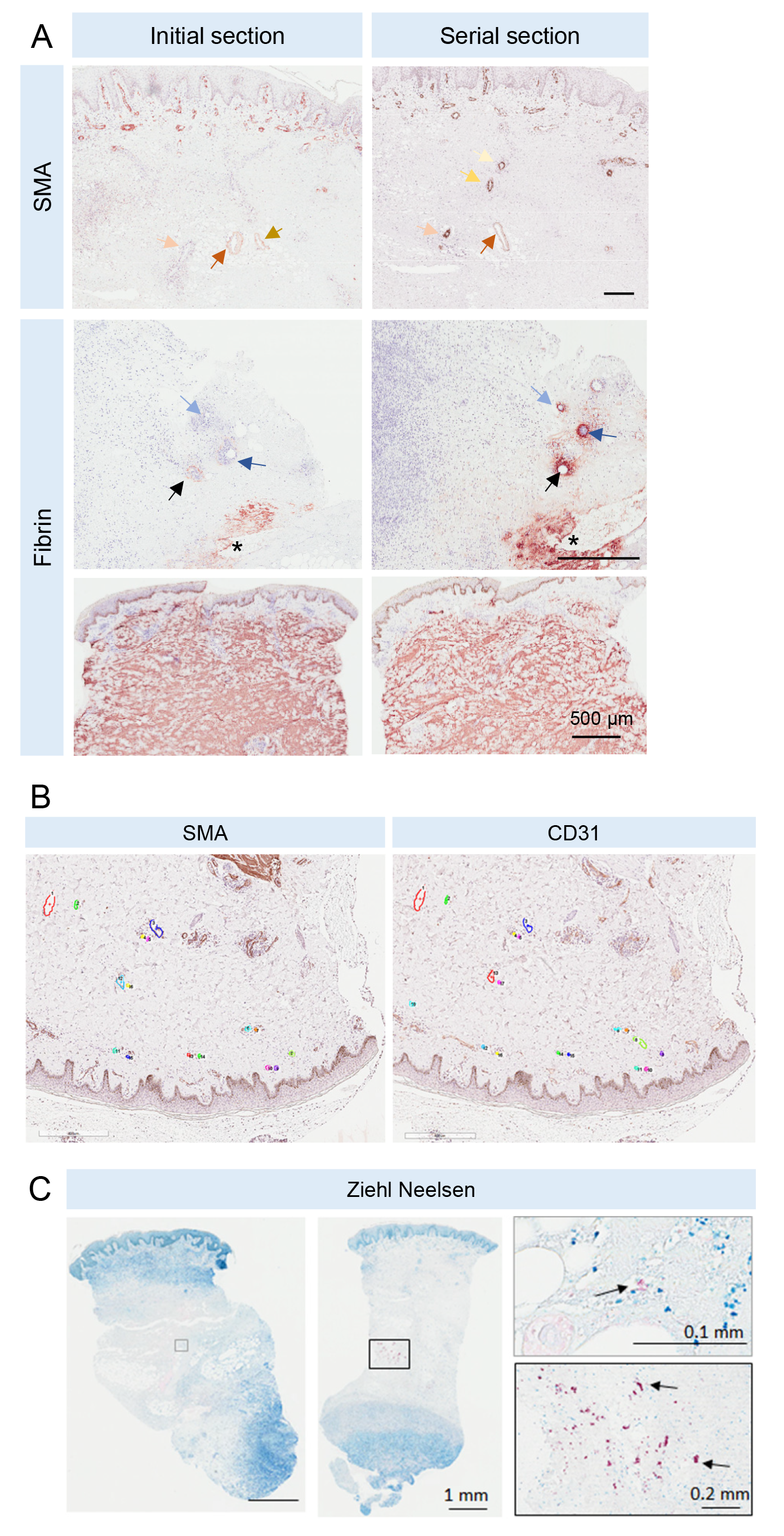

Supplement: S2 Fig — A. Comparison of the staining patterns with the same anti-SMA or anti-fibrin antibody and conditions seen for the same punch biopsies at different positions in the tissue block. The “initial section” displays those performed for Ogbechi et al., 2015 [42], whereas the “contiguous section” was performed for the present work. Arrows in different colours label the same vessel identified in different tissue sections. Scale bar = 500 μm. Note how the vessel phenotype can vary even over small distances. B. An example of vessel identification and labelling (in colours with individual number indicated) in serial tissue sections stained with anti-SMA and CD31 antibody. C. Mycobacterial clusters (in purple, indicated with arrows) are identified in histological sections from 2 BU patient punch biopsies with Ziehl-Neelsen staining. Scar bars as indicated. (TIF) [file ppat.1010280.s002.tif]

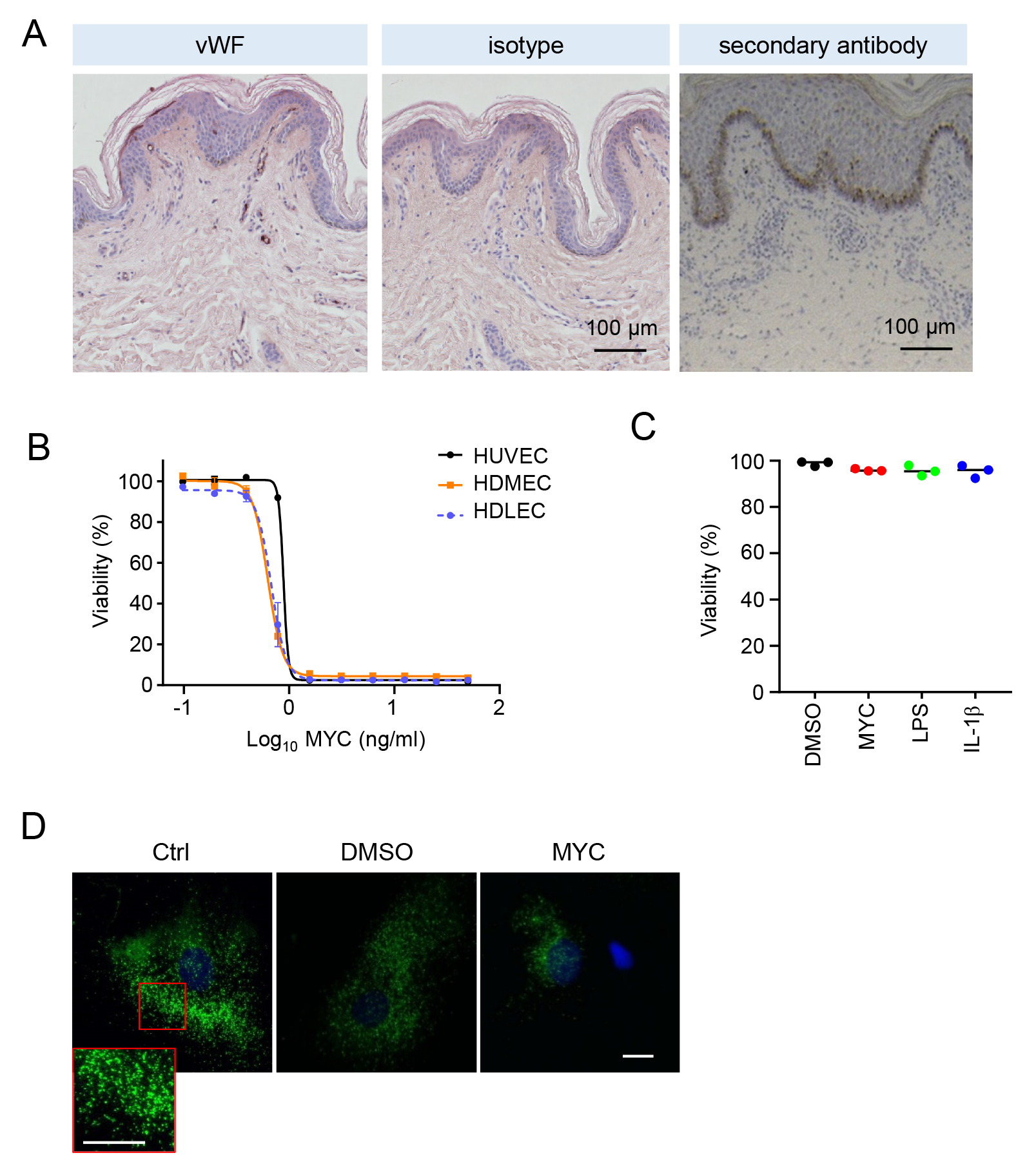

Supplement: S3 Fig — A. Histological sections of a healthy subject or BU patient stained with anti-vWF antibody, the respective isotype control or secondary antibody alone and counterstained with Haematoxylin. B. Cell viability of HDLECs, HDMECs and HUVECs exposed to a variety doses of mycolactone for 5 days was determined using alamarBlue assay and presented as a % where 100% is the value obtained from cells treated with solvent control DMSO. C. Viability of HUVECs that were untreated or exposed to 0.02% DMSO, 10 ng/mL mycolactone (MYC), 100 ng/mL IL-1β or 400 ng/mL LPS for 24 hrs using CellEvent detection kit. The number of active caspase 3/7 and PI-positive cells were counted per field and expressed as a % of total number of cells. Three different fields representing the top, bottom and middle part of plate were taken. D. HDMECs were treated with 10 ng/mL of mycolactone (MYC), 0.02% DMSO or untreated (Ctrl) for 24 hours. Cells were fixed, permeabilised and immunostained with anti-vWF antibody. vWF-containing granules are shown in green and nuclei stained with DAPI (blue). Scale bar = 20 μm. (TIF) [file ppat.1010280.s003.tif]

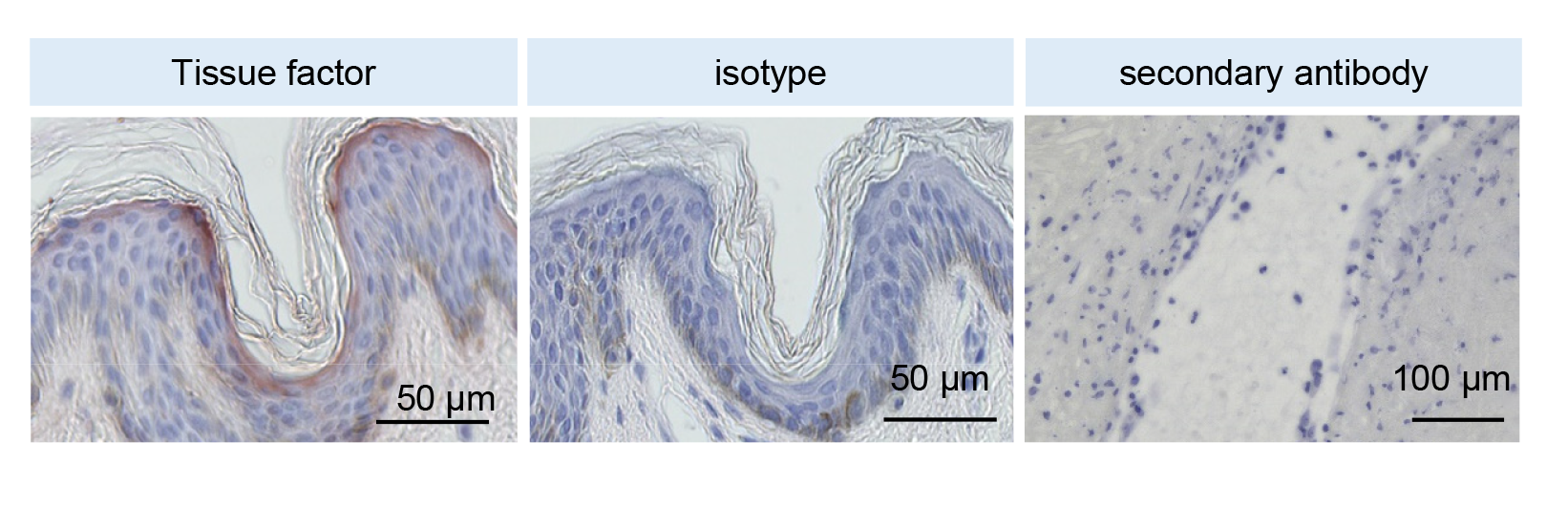

Supplement: S4 Fig — Histological sections of a healthy subject or BU patient stained with anti-TF antibody, the respective isotype control or secondary antibody alone and counterstained with Haematoxylin. Scale bar as indicated. (TIF) [file ppat.1010280.s004.tif]

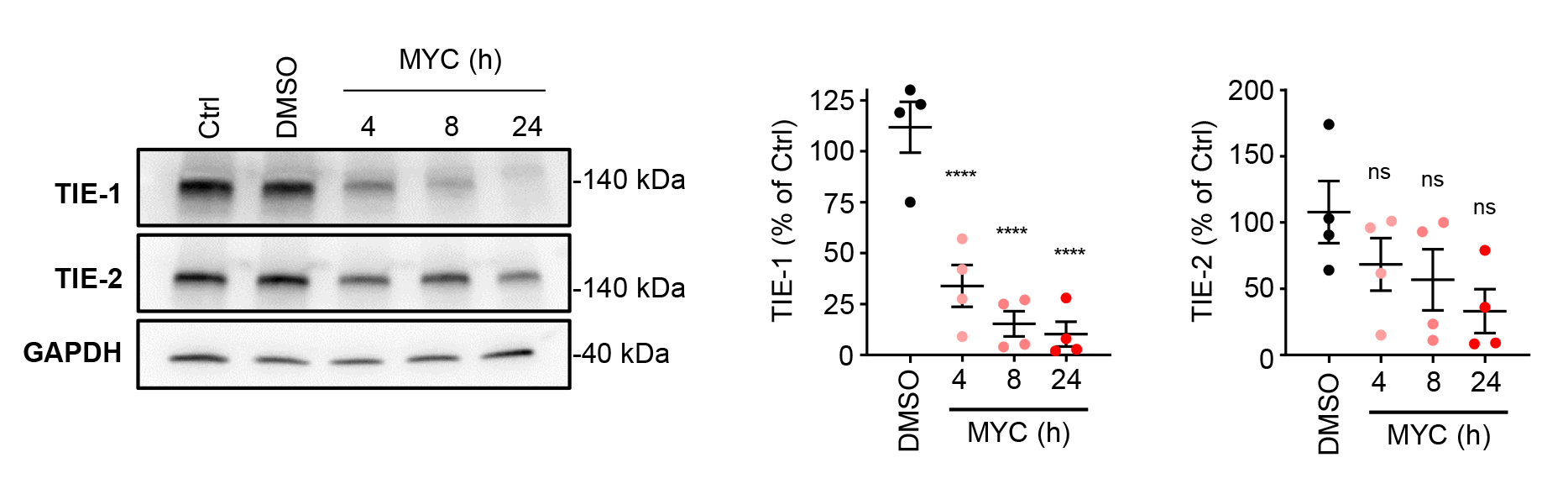

Supplement: S5 Fig — HDMECs were exposed to 0.02% DMSO, 10 ng/mL mycolactone (MYC) or remained untreated for the indicated times, lysed, and subjected to immunoblotting. Immunoblot intensity of TIE-1 and TIE-2 was normalised according to GAPDH and expressed relative to untreated control. Values represent the mean of three independent experiments ± SEM. ns, not significant; ****, P < 0.0001. (TIF) [file ppat.1010280.s005.tif]
